# Switching between reading tasks leads to phase-transitions in reading times in L1 and L2 readers

**DOI:** 10.1371/journal.pone.0211502

**Published:** 2019-02-05

**Authors:** Sebastian Wallot, Jun Taek Lee, Damian G. Kelty-Stephen

**Affiliations:** 1 Max Planck Institute for Empirical Aesthetics, Frankfurt, Germany; 2 Interacting Minds Centre, Aarhus University, Aarhus, Denmark; 3 Department of Psychology, Grinnell College, Grinnell, Iowa, United States of America; University of Texas at El Paso, UNITED STATES

## Abstract

Reading research uses different tasks to investigate different levels of the reading process, such as word recognition, syntactic parsing, or semantic integration. It seems to be tacitly assumed that the underlying cognitive process that constitute reading are stable across those tasks. However, nothing is known about what happens when readers switch from one reading task to another. The stability assumptions of the reading process suggest that the cognitive system resolves this switching between two tasks quickly. Here, we present an alternative language-game hypothesis (LGH) of reading that begins by treating reading as a softly-assembled process and that assumes, instead of stability, context-sensitive flexibility of the reading process. LGH predicts that switching between two reading tasks leads to longer lasting phase-transition like patterns in the reading process. Using the nonlinear-dynamical tool of recurrence quantification analysis, we test these predictions by examining series of individual word reading times in self-paced reading tasks where native (L1) and second language readers (L2) transition between random word and ordered text reading tasks. We find consistent evidence for phase-transitions in the reading times when readers switch from ordered text to random-word reading, but we find mixed evidence when readers transition from random-word to ordered-text reading. In the latter case, L2 readers show moderately stronger signs for phase-transitions compared to L1 readers, suggesting that familiarity with a language influences whether and how such transitions occur. The results provide evidence for LGH and suggest that the cognitive processes underlying reading are not fully stable across tasks but exhibit soft-assembly in the interaction between task and reader characteristics.

## Introduction

Research on reading has identified multiple levels at which the reading process operates, from the perceptual front-end to the integration and processing of higher-level semantic information. While there are competing hypotheses on what might constitute a particular level or sub-level, the most prominent ones are word identification, syntactic parsing, and higher-level semantic integration [1–4]. There is plenty of evidence for distinct processes that operate at these different levels, but the investigation of these three levels constitutes almost three different research areas. Research on these different levels is often distinguished by the use of different designs, manipulations, and preferred types of stimuli. For example, investigations of word recognition heavily rely on paradigms using individual words or sublexical features, for the most part not extending the scale of presenting two related words. Word-recognition researchers prefer particular tasks such as word naming or lexical decision. However, some studies of eye movements during reading might also be classified as primarily aiming at word recognition processes because eye movements are often modelled as to be primarily driven by word-level features (e.g., [5,6] but see [7]). Investigations of syntactic parsing prefer a sentence level scale, with studies often not extending beyond the scale of two sentences, because one sentence alone already provides most of the linguistic space needed to investigate syntactic structures [1]. Here particularly, eye tracking and EEG-recordings are prominent, even though self-paced reading has been used as well. Finally, research on higher-level semantic integration necessarily needs to investigate longer text passages where the build-up of an overarching meaning is possible (e.g., into situation models), and the reading process is mostly measured using eye movements or self-paced reading.

Naturally, reading of connected texts relies on all of these levels working in concert—not just in parallel but with discourse, sentence, and word processing all having the opportunity to inform one another. However, because of these methodological disparities, research on reading across these three levels is seldomly integrated. Connections between research findings on the different levels usually do not go much further than incorporating prominent variables that have been primarilty investigated at one level within one paradigm as covariates when investigating another level. To our knowledge, the most far-reaching transfer across these levels is the integration of variables such as word frequency, word length or sentence length as covariates in paradigms that investigate connected text reading (e.g., [8–12]). Accordingly, not much is known about what happens to the reading process when crossing the bridge between different reading tasks that either differ in their emphasis on one of these levels or simply do not contain a particular kind of information on which some of these levels operate (i.e., reading a random word list does neither afford syntactic parsing, nor higher-level semantic integration).

However, judging by the practice of transferring covariates between research of these different levels, the tacit assumption seems to be that those levels *always* participate in the reading process in the way they have been observed within their specific research paradigms. But that tacit assumption is at odds with the empirical evidence. Upsetting this covariate-based confidence of sameness of effects across levels is recent research on the word-length and frequency effects: Effects of word frequency and of word length actually decline steadily across the continued reading of connected texts [13] and the effects of word-frequency actually increase over the course of a lexical decision task [14].

The latent assumption of this parsing of levels and transferring of covariates across them is twofold: first, that sentence reading is effectively a combination of word identification and syntactic parsing and second, that text reading is a combination of word identification, syntactic parsing, and higher-level semantic integration. No matter how much a particular researcher agrees with this assumption, it can safely be said that we know little about what happens at the boundary between reading tasks that primarily afford processing on one of the levels and reading tasks that afford processing another level. However, the differences between these methodologies suggest that the boundary is sharp and that participants must transition from one task to the next very quickly. While there are occasional studies discarding the first couple of reading times to remove any task-onset effects, there is no such general practice in research on reading. Hence, the assumption seems to be that, following proper instruction of participants, the reading process performs relatively stably in a specific mode that encompasses a certain combination of processes on the different levels within a very short time-interval, and responses right from the start of stimulus presentation generate valid data points (fixation durations, word- or sentence reading times, etc.).

Outlining the position so far, the current paper focuses on the question of what happens at the boundary of two reading tasks, specifically the boundary between levels of word-reading and connected-text reading. We want to outline an alternative hypothesis about the impact of task switching that predicts a discontinuous and potentially long-lasting effect of switching between different reading tasks and so between different processes of reading. As summarized above, current practices with reading data suggest that the reading process should be stable across these levels. Hence, a reasonable baseline expectation for the impact of task switching is that any impact of switching from reading at one level to another should be short-lived and additive. Our alternative hypothesis is the Language Game Hypothesis (LGH) of reading aimed at testing the prediction that switching from one reading task to another will lead to phase transition-like patterns in reading process measures [15]. In the following sections, we will describe LGH in three major veins: its conceptualization of reading as an activity, its prediction that task-switching will lead to phase-transitions in the reading process, and its implications of specific consequences of phase-transitions in the context of reading. We will test LGH in two studies of how readers—both native English speakers (L1) and readers with English as their second language (L2)—switch between reading of random word lists and reading of ordered texts using self-paced reading.

### The language game hypothesis (LGH) of reading: an introduction and background

As the name implies, LGH was inspired by Wittgenstein’s concept of the “language game” [16]. The idea of a language game is that the use of language in a particular situation adheres to “rules,” but those rules are contingent on properties of the context and the interlocutors, as well as cultural-historical influences. What the language game conception has in common with more widely held assumptions in (psycho-)linguistics and reading research is that language-use is governed by rules. Where it differs from this assumption is that these rules are not primarily linguistic in nature, and that they are not universal in the sense that they reveal the basic structure that governs language-use across all or most situations. Rather, there are different language games, some of which might have similar rules that govern them, and some might have radically different rules. LGH adapts the notion of the language game to reading, specifically interpreting different reading tasks and/or stimulus configurations in analogy to different situations of language-use in which the perceptual-cognitive processes of reading unfold. Accordingly, different reading tasks might be governed by very different “rules” and not by a universal reading process [17]. LGH was formulated to address concerns regarding the problem of meaning in contemporary reading research, but also with regard to mounting evidence that linguistic rules are not universal across different languages and reading tasks (e.g., [18]). On the contrary, the evidence indicates that the perceptual-cognitive processes behind reading are similarly co-constituted by characteristics of the reading task or material, that is they are not rigid but softly-assembled processes. In the following sections, we will discuss each of these problems and end with specific predictions of LGH for the case of switching between two reading tasks.

#### Concern over cognitive architecture: Rigid or soft assembly?

Our choices for assumptions about cognitive architecture are twofold, at least: cognitive processes governing reading (and other mental feats) could be rigidly assembled, conforming to a set of context-independent processes that work according to a fixed set of rules, or they could be softly assembled, conforming to emergent properties resulting from self-organizing processes founded on multi-scaled interaction of organisms with situational properties [19,20].

Perspectives approaching the mind as a self-organizing process rest on the foundations of higher-level interaction effects between experimental-psychological manipulations [21,22] and, more profoundly, by the observation of fractal and multifractal patterns in behavioral and (neuro-)physiological measures [23], some of which actually affect perceptual and cognitive outcomes [24–38].

On the one hand, higher-level interactions seem like they might clutter our view of the basic properties of mental processes, those properties that we might need for handling models of the different aspects of human cognition, action and perception (e.g., “the degrees of freedom problem”; [39,40]). On the other hand, the seeming clutter might reflect a higher-order structure that we ignore at our theoretical peril. The (multi-)fractal patterns in human cognitive performance—even in reading of text or random word lists—suggest in a mathematically estimable and empirically tangible way that measures of human behavior may not always decompose into different, distinguishable sources of variability, but that the presumed different and independent components of that behavior are inextricably woven into each other [23]. These findings have invited the interpretation that human cognition and behavior are self-organizing processes that support the emergence of higher-level functions (such as word recognition, syntactic parsing, or semantic integration during reading), but this theoretical approach entails that those functions are a temporary form of organization that comes about in the interaction between an organism and context, and are not universal.

The situational roots entailed by the LGH suggest that measures of cognitive performance should exhibit complexity properties when changes in situation or task occur, which otherwise would be ascribed solely to the cognitive processes themselves [15,17]. They further suggest that so-called “complexity flags” (e.g., changes in fractal properties and indicators of phase-transition outlined in more detail below) are more fundamental basic properties that we might expect from self-organized cognitive architectures [20,41,42].

#### The question of meaning in particular

The second concern is that the concept of meaning itself is not much discussed or outlined in reading research (or cognitive psychology in general; [43]). It is usually taken for granted in the definition of what reading is, that some form of information is present that can be understood and interpreted by a reader and has some kind of impact on the reader’s (potential) behavior. Indeed, the whole premise of a logical coherence taking on causal impact in shaping behavior may be one of the most stunning and ambitious possibilities distinguishing the innovations of cognitive science against any other science [44].

The majority of theories and models of reading seem to conceptualize meaning based on Frege’s axiom of composability [45], where the (literal) meaning of a sentence is composed of the meaning of its constituent words in their syntactical arrangement. After all, these theories and models derive or ascribe all the driving components of the reading process from or to the text material, quantified by variables such as word length, word frequency, word class, word order, sentence coherence characteristics and so forth. Here, successfully understanding a word is the mapping of an external percept to its mental representation anchored on word characteristics. On this basis, syntactic structure provides a means for combining these elemental word meanings whose combination supports a reader’s judgment of coherence within or across sentences. In order for this mapping process to work (i.e., for word and text characteristics to be effective drivers of the reading process), elements on both sides of this relationship need to be stable.

However, this requirement comes at the cost of having to define meaning in such a way that it is internal to the cognitive system, where the blueprint for reading research is the concept of the mental lexicon. Here the meaning of one word is defined in terms of other words or defined in terms of graded associations to many, potentially all other words [46]. Again, such a conceptualization of the mental lexicon must be rigid to fulfill its purpose, namely, to preserve the meaning of a word. More pressingly, this definition renders language meaningless, because it is fully tautological [16].

Let us consider a brief example of a mental lexicon containing a finite set of words, for example *wood*, *tree*, and *bench*. As long as this lexicon requires the definition of meaning of those words to be stable and self-contained within the lexicon, any attempt to define their meaning leads to a tautology. For example, we could define *wood* = : *tree*, and *bench* = : *wood*, but what about *tree*? If we leave *tree* undefined, so are *wood* and *bench*. If we define *tree* by *wood* or *bench*, then it implies nothing more that *tree* is *tree*. This problem can be solved if one allows other factors to co-define meaning, such as properties of the state of the organism or the situation at hand. However, this necessarily implies that meaning–and the relation of the word to each other–changes as organisms and situational properties change. To summarize, while we define reading as an activity that is about meaning, our conceptions of what drives the reading process are at odds with what we would require of a meaningful language. Beside these conceptual considerations, empirical evidence casts a shadow over Fregean stability for the sake of Fregean composability: the most basic of these text-characteristic predictors at the word level (e.g., word length and word frequency) are not stable across the duration of different reading tasks [13,14], and also situation-model dimensions from discourse-level theories fail to show consistent effects when applied to long texts [8].

The motivation then for the LGH was to relax some of the requirements about the definition of meaning. As this required also the relaxation of the assumption of rigidity of the underlying processes, founding an ontology of the mental architecture in softly-assembled processes of self-organization seemed a natural grounding.

#### Language-game hypothesis proposes readers draw a softly-assembled context-dependent meaning from the printed word(s)

Hence, the language-game conception relaxes the need of defining meaning strictly in terms of stable linguistic constituents and entertains the idea that various proximal and remote non-linguistic factors co-define meaning as well. From the perspective of the self-organizing nature of cognitive processes–and hence also those processes that participate in reading–something like LGH becomes even a necessary point of departure because self-organizing processes rest on interactions operating across many scales at once [47–50]. Such a move also avoids the problems of the mental-lexicon restatement-rather-than-solution tautology and the need for stable relationship between linguistic features of language and cognitive performance, the former being primarily a problem of logical inconsistency and the latter being a problem of empirical findings that are inconsistent with the supposed stability of the relationship between linguistic characteristics and reading performance [13,14].

Besides sidestepping some of the critical problems associated with the current definition of meaning in reading research, the language-game hypothesis generates novel research questions. For example, what non-linguistic aspects co-define meaning in language? And how exactly can linguistic and non-linguistic information be combined? The current research is part of an effort to begin identifying the critical links between linguistic and non-linguistic constituents of language and meaning.

Rather than assuming rigidly-assembled mapping rules from linguistic characteristics to meaning, the LGH proposes that such mappings cannot be completely defined on the linguistic level and depend as well on temporal or situational characteristics of a reading task. That is, manipulating the presentation and the responses available to a reader should yield reading tasks that seem to tap into the same context-independent cognitive processes but effectively prompt a different reading process or bring into relief different systematicities in the “same” reading process. Changing the reading situation can entail changes in how the reading process unfolds over time. LGH specifically predicts that “reading games” (here: two reading tasks) that are sufficiently different from another lead to qualitative changes in the reading process, not merely quantitative ones.

For present purposes, we consider a contrast between reading of random word lists and connected text. A strict compositional account would construe the difference between reading ordered text and reading random-word lists as an additive difference: wherein context-independent characteristics of word identification are present in the same way in both tasks, but the ordered text reading task adds a new context-independent syntactic parsing process that language structured in sentences affords. On the other hand, approaching reading as a thoroughly context-sensitive process, LGH predicts that the cognitive processes involved in reading need time to softly-assemble to the cognitively relevant structure of different reading tasks. That is, a change in reading task (i.e., switching from random word list reading to connected text reading) will prompt a re-assembly of the cognitive architecture to the new task. Particularly, the hypothesis is that this adaptation takes time because the structure of the new task is unknown and so requires the cognitive system to explore in order to accomplish a novel assembly.

We propose to test this hypothesis by using the non-linear dynamical tool of recurrence quantification to diagnose whether the transition from one reading task to another resembles a nonlinear phase transition. Conceptually and algorithmically, we follow in the footsteps of earlier work in which nonlinear recurrence properties of participants’ finger [42] and eye movement [51] dynamics during a visuospatial reasoning task helped to predict the discovery of qualitatively new strategies. In the next section, we provide an example of such a nonlinear phase-transition through a simplified and well-documented model nonlinear-dynamical system. Our test of the LGH in reading will then apply this recurrence-quantification tool to a series of word-reading times to assess nonlinear-dynamical stability across the switching of a reading task.

### Capturing phase-transitions using recurrence quantification analysis (RQA)

A classical model system for the analysis of nonlinear phase-transitions is the Lorenz system [52]. The Lorenz system is a dynamical system composed of three coupled ordinary differential equations (see Eq 1) and was originally invented for the purpose of investigating long-term atmospheric forecasting:

$$\begin{array}{l} \dot{x}=\sigma \left(y-x\right)\\ \dot{y}=\left(\rho -z\right)-y\\ \dot{z}=xy-\beta z \end{array}$$ (1)

**Fig 1:**
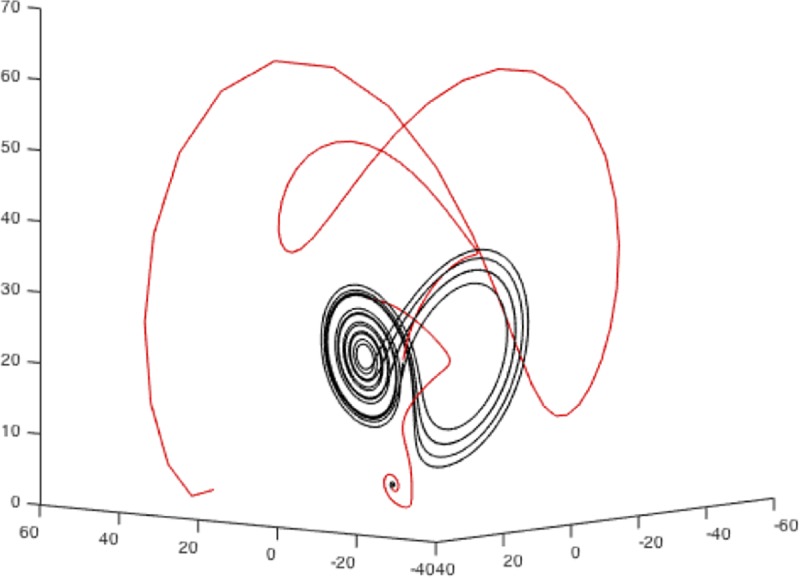
Example of the Lorenz system transitioning between two stable phases. Stable phases appear in black, and transitions appear in red.

The system can exhibit two qualitative kinds of behavior, one attractor where the system behaves chaotic (i.e., the “butterfly”-shaped loops) and one tightly converging orbit where the dynamics on the three dimensions tend towards a single focal point over time. Changing the parameter values can force the system from one phase (e.g., the “butterfly” attractor) to another (i.e., the single-focus orbit). Fig 1 illustrates this with the two states–the “butterfly” attractor and the single-focus orbit–in black, and the initial transition into the “butterfly” attractor, as well as the phase-transition from “butterfly” to single-focus orbit attractor in red.

Hence, phase transitions in dynamical systems such as the Lorenz system are brought about by changing the parameters in the equations, which can be done gradually or abruptly. As we notice, changing the parameters abruptly (as we have done in the example presented in Fig 1) does not lead to an abrupt change in the dynamics of the system, but invokes a phase-transition, where the system exhibits a behavior that connects the two phases, but this behavior neither draws a straight path between the two, nor is the transition phase a composition of these two.

Overall, this situation is a phenomenologically useful model for what we expect to happen at the switch-point between two reading tasks. The parameters in the Lorenz system are analogous with manipulations of a reading task, where parameters are held constant for a while and lead to the display of one kind of dynamics in analogy to the random-word list reading task, and the abrupt change to a new steady parameter state would be analogous to the onset of the ordered text reading task from one word to the next. Behaviorally, we see two different kinds of regularities in the two phases of the Lorenz attractor that may be analogous to the potentially different dynamics of random word list versus connected text reading. The phase-transition in the dynamics that connects the two phases would be analogous to the re-organization of the cognitive processes when switching from one task to another. Note, however, that we are primarily interested in the qualitative transitioning behavior that the Lorenz system displays (two phases and a nonlinear phase-transition) in analogy to our investigation of switching between reading tasks. We do not mean to imply that the Lorenz system itself is in anyway a good underlying model for the process of reading—or of anything but atmospheric flows as originally intended.

Next, in order to capture such phase-transitions quantitatively, we can subject the data observed from the Lorenz system to recurrence quantification analysis (RQA), which has been used as a tool to analyze dynamics systems [53]. As the name implies, the core-concept of this analysis is *recurrence*–repetition of elements in a sequence or time series. The core tool of the analysis is the recurrence plot (RP), which is a means of displaying and charting repetitions in a sequence. As we will see further below, the analysis is usually not performed on the original 1-dimensional sequence or time series, but on its phase-space portrait embedded in a higher-dimensional space (such as the display of the three-dimensional dynamics of the Lorenz system in Fig 1).

**Fig 2:**
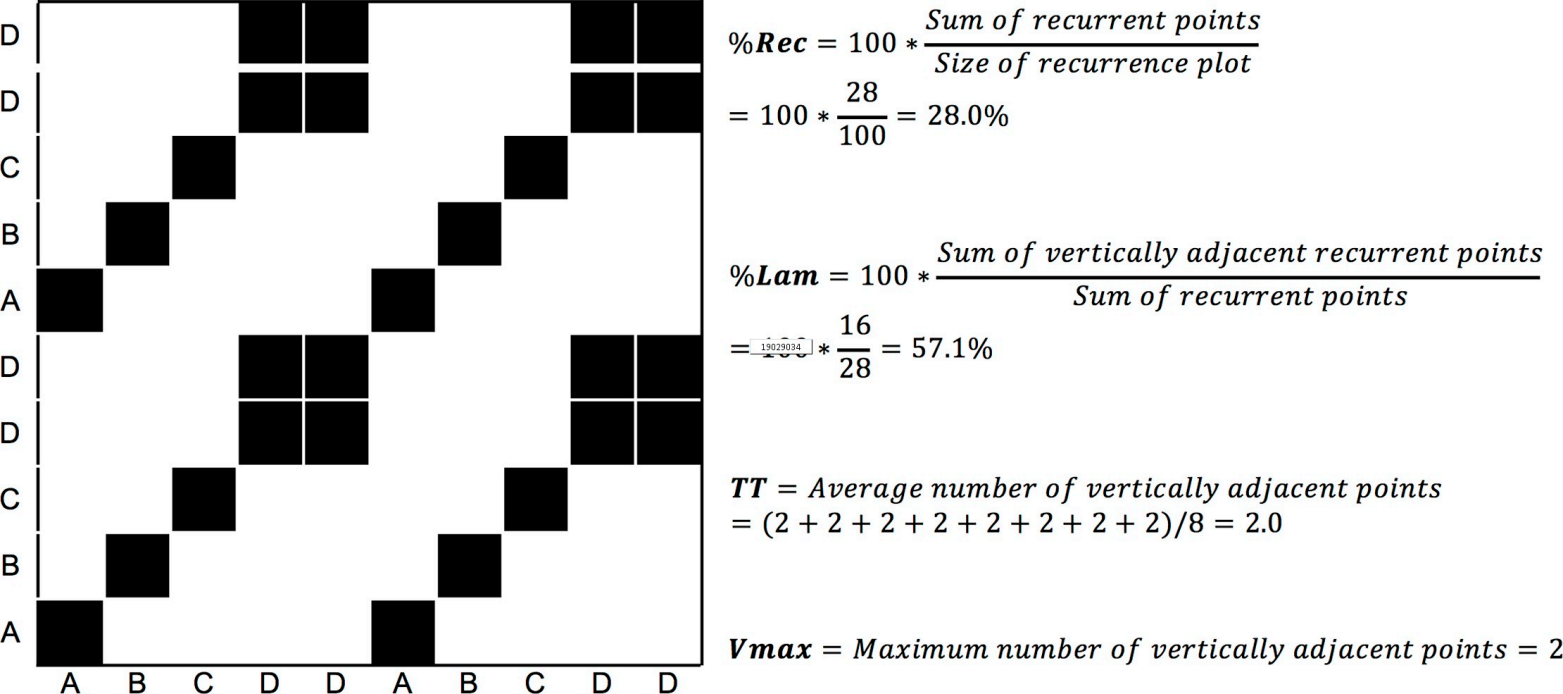
Example recurrence plot (RP). The black squares indicate recurrences within the sequence “ABCDDABCDD”, plotted on the x- and y-axes of the RP. To the right of the plot are the calculations of some of the recurrence measures in this example.

We want to introduce the analysis briefly, using an example adapted from [54], using a simple, short 1-dimensional nominal sequence, “ABCDDABCDD”. The sequence is arbitrary (it could represent a series of fixations to different regions of interest during a reading task), but is does not appear to be random, containing similar repetitive sub-sequences. A recurrence plot can be used to visualize these repeating characteristics by comparing all the elements of such a sequence with themselves, when aligned in the two dimensions of the plot (Fig 2).

The RP is not just a useful tool to visualize the sequential correlations in a sequence *but can be used to quantify their auto-correlation properties*. For example, the sum of all recurrent points on the plot tells us something about the repetitiveness of the individual elements in the sequence, and we refer to this quantity as percent recurrence (*%REC*). Counting all recurrent points that have vertically adjacent recurrent points and dividing them by *%REC* tells us something about the degree to which adjacent elements in the sequence stay the same or whether the dynamics of a time series stay in the same state. This quantity is called percent laminarity (*%LAM*). Counting the average length of all vertical lines on the RP quantifies the average size or duration of such states (the mean vertical line length know as trapping time, *TT*), counting the longest vertical line on the RP quantifies the size or duration of the longest state (the maximum vertical line length, *maxV*). However, there are many more ways to quantify the RP, and all of them provide potentially different information about the dynamics of a sequence or time series. Table 1 summarizes the recurrence measures and their definitions that we are using in this paper.

**Table 1:** Summary of recurrence measures used in this study.

Variable Name	Definition	Quantifies…
Percent Recurrence (%*REC*)	Sum of recurrent points in RP /Size of RP	…repetition of elements across the two sequences.
Percent Laminarity (%*LAM*)	Sum of vertically adjacent recurrent points / Sum of recurrent points in RP	…how many of the individual repetitions co-occur in connected states.
Trapping Time (*TT*)	Average length of vertical lines in RP	…how long the average duration of a connected state is.
Maximum Vertical Line Length (*maxV*)	Length of longest vertical line in RP	…how long the longest duration of a connected state is.

*Note*. For the description of additional measure, see for example Marwan et al. [53].

**Fig 3:**
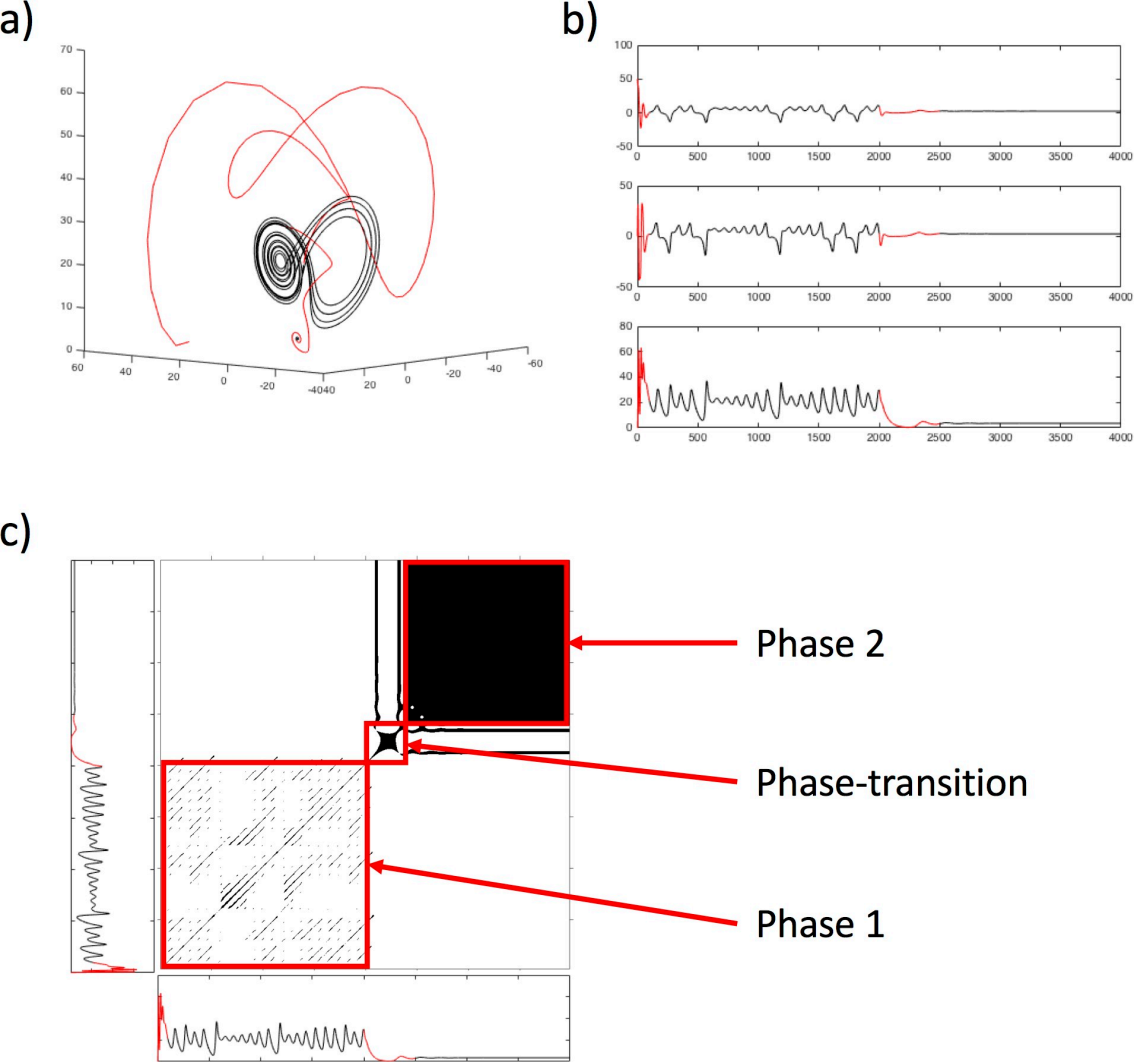
Recurrence plot or the Lorenz system through a transition. The Lorenz system with phase-transitions (a), the values on the respective three dimensions (b) and the RP based on the data of the z-dimension (c), indicating the two stable phases as well as the transition between them.

An RP can also be obtained for the dynamics of the Lorenz system displayed in Fig 1. To that end, we pick one of the three dimensions of the Lorenz system (here: the z-dimension), which provides us with a 1-dimensional time series of x-coordinate values of the systems behavior, similar to the 1-dimensional time series of word reading times that we are analyzing in order to investigate the effects of switching from random word reading to ordered text reading. Fig 3 presents the 3-dimensional dynamics of the Lorenz system (Fig 3A) together with the individual data from each of the three dimensions (Fig 3B), the lowest of which is represented as a recurrence plot (Fig 3C).

**Fig 4:**
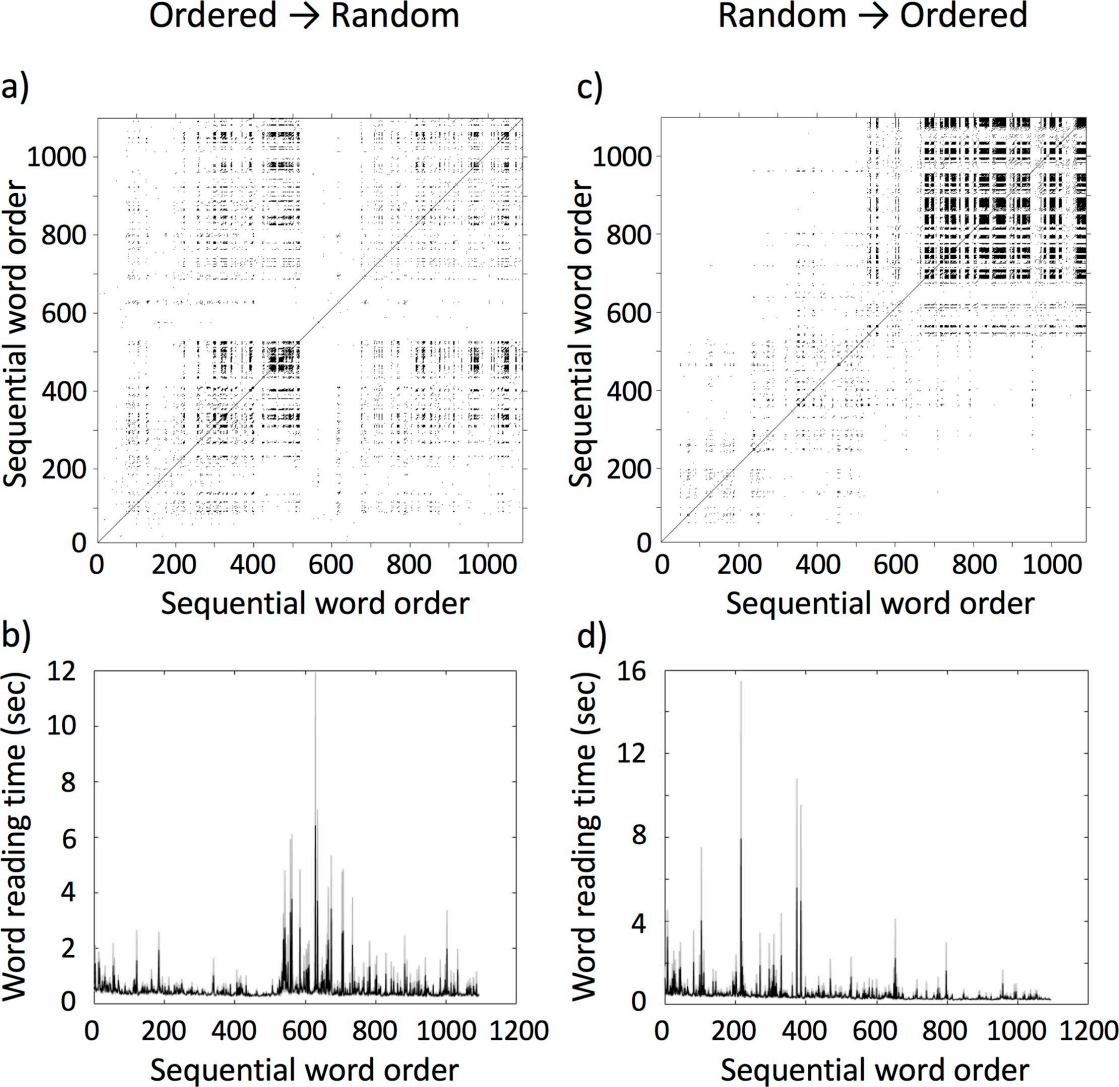
Example recurrence plots and word reading series. Average RP of the O→R condition (a) and the associated average time-series of word reading times (averages in black and standard deviations in grey) (b), and average RP of the R→O condition (c) and the associated average time-series of word reading times (averages in black and standard deviations in grey) (d) for Study 1.

As can be seen, the RP captures the different dynamics of the Lorenz system–the first phase where the system exhibits attractor dynamics, but also the second phase where the system converges to a single-focus orbit. Moreover, we can see the transition phase in between where the system initially drops in terms of recurrences, and then starts to settle into the single-focus-orbit attractor. Such changes can be quantified by examining the change of the RQA measures over time and are indicative of whether a system undergoes a phase-transition [55,56]. Note, however, that the dynamics of the Lorenz system are better captured by measures that quantify diagonal lines on the RP, while the reading time data that we are interested in modelling are better captured by the vertical line measures that we described above. This is so because sequences of reading times do not exhibit well-defined trajectories as the Lorenz system, whose recurrences appear as diagonal line structures on an RP (see Fig 3B), but rather exhibit a clustering of adjacent recurrence points, evident as patches of recurrence on the RP (see Fig 4 below).

### Summary of hypotheses

To summarize, contemporary theory and practice in reading research suggests that the different processes composing reading are fairly context-independent across tasks and that the cognitive system is able to start processing language stably during reading within a handful of words at the most. Even though contemporary theories are silent on what would be expected to happen at the switch-point from one reading task to another, the above summary suggests that switching from random word list reading to connected text reading is quickly resolved by the cognitive system.

From the perspective of LGH, there is no rigid matching between stimulus characteristics and their mental representations, and the nature of processing of text material during reading is inherently dependent on task and non-local stimulus characteristics. The cognitive system adapts to such non-linguistic situational aspects in order to processes written language, and switching between two different reading tasks entails a change in the coordination of cognitive processes, not merely an addition of one level of processing to another. Hence, we expect a pattern in the reading process after the switch point that is similar to nonlinear phase-transition in dynamics systems and is indicative of more global adaptation of the cognitive system to a task. Hence, there are three patterns of results that relate to the different hypotheses:

H1: The reading process is completely stable across random word list and connected text reading and quickly adds new relevant levels of processing after a change in tasks–a minor disruption of switching from one reading task to another on the order of a hand full of words or less.
H2: An adjustment of the viewpoint represented by H1 to respect timing differences due to the two different reading tasks. The reading process is stable at the different participating levels, but because different reading tasks recruit different levels of processing, the reading time dynamics differ between the two reading tasks. Nevertheless, switching from one task to another happens quickly as it is only a matter of enlisting a new level of processing.
H3: The reading process is inherently co-dependent on task and stimulus characteristics and hence switching from one task to the other entails a cognitive reorganization of the reading process that leads to a longer-lasting adaptive process akin to a nonlinear phase-transition. This is the prediction based on LGH.

## Study 1

The aim of study 1 was to test the outlined hypotheses of the presence of a phase-transition in the reading process between two reading tasks, namely random word list reading and ordered text reading. To that end, participants performed a self-paced reading task in which they were randomly assigned either to read a sequence of random words and then subsequently to read ordered text half-way through the task or, in another condition, to read words appearing as ordered text and then, half way through the task, to read words in random order.

We did not forewarn participants of this change in stimulus presentation. Participants simply continued to read while the timing of consecutive button presses to reveal each new word provided an estimate for the reading time of each new word. We then used recurrence quantification to analyze the series of word reading times to test for phase-transition properties around the half-way point when the task changed. In analogy with the Lorenz system, we expect a drop in repetetive structure of consecutive reading times after task switching. Hence, we expect lower values for RQA measures that capture repetitiveness, such as lower *%REC*, but particularly lower *%LAM*, lower *TT*, and lower *maxV*, as these three measures capture the overarching degree of temporal clustering over many reading times.

## Materials and methods

### Participants

Thirty participants, undergraduate students of Grinnell College, IA, USA (average age = 18.26, SD = 1.22, ranging from 18 to 24 yrs.; 20 were female) participated in the study. The words and the text were presented in English, and all participants were native speakers (L1) of English and all had normal or corrected-to-normal vision.

### Ethics approval

The study was approved by the Grinnell College Institutional Review Board.

### Materials and apparatus

The text presented to participants was taken from the children’s book Alien and Possum: Hanging Out by Tony Johnston [57] describing the adventures of two fictional characters. We used a slightly modified version of the text adapted from O’Brien and colleagues [58], containing 1082 words, who used the text to assess silent-reading fluency in school children and young adults. Also, none of the illustrations in the original book appeared with the text. The text was displayed on a Dell desktop PC, with a custom script (MatLab-Psychophysicstoolbox; [59]) running the text presentation software. Two version of this text were prepared. In one version, R→O, the first 529 words were randomized within each sentence, while the second part of the story, equaling 554 words, was left in its original order. In the other version, O→R, the first 529 words were left in the original order, while the second part of the story with the remaining 554 words was randomized within sentences.

The cut-off of 529 words for splitting the story was chosen because it contained roughly half the words of the whole text and because it marked a split point in the text after which a new display of text began. The words were randomized within sentences (instead of, for example, across the whole first or second part of the story) in order to keep the local distribution of word characteristics similar across the same randomized and ordered text version. Moreover, piloting of the stimuli by the experimenters and by research assistants who were not aware of the manipulation suggested that the words so re-arranged were still perceived as a sequence of random words.

### Procedure

Participants were randomly assigned to either one of the two conditions, R→O or O→R, so that 15 participants performed in each condition. Written consent was obtained from all participants. Participants were seated in front of a computer monitor and were told that their task would be to read words on a monitor until a message appeared that the study was over. Specifically, participants were told that their task was to press a button in order to reveal a word that would appear on the computer monitor, then read that word, and then press the button to make the next word appear after they were done reading that word. There was no mentioning of either the words appearing in a specific order, or any changes of the stimuli throughout the task. Thereafter, participants received a short test trial where they could familiarize themselves with the self-paced reading procedure, after which the experimental task started. The experiment ended with a message on the screen that the reading task was over. Then, participants filled in a few demographic questions and were finally debriefed and received partial credit towards a research-experience grade in introductory psychology for their participation. The reading task took at about 10–20 minutes, depending on the individual reading speed. The intervals between consecutive button presses in the self-paced reading task were treated as estimators of word reading time and were further analyzed as described below.

### Data analysis

As described above, we used RQA to evaluate the presence (or absence) of patterns in reading times that are indicative of phase-transition-like behavior at the switch point between reading tasks. In order to do so, however, we need to quantify *changes* in the recurrence measures at the switch point, and hence cannot calculate recurrence measure globally across the whole reading time series for each participant. Rather we need to calculate recurrence measures for sub-windows of the reading time series per participant, effectively transforming the time series of reading times to a time series of recurrence properties. We did this by splitting the original series of 1082 reading times into 107 windows of 20 data points, overlapping by 10 data points, calculated an RP for each sub-window, and accordingly obtained the RQA measures *%REC*, *%LAM*, *TT*, and *maxV* for each sub-window. Vertical-line based measure were used, because the dynamics of reading times do not exhibit well-defined trajectories as the Lorenz systems (see Fig 3B), but rather exhibit a clustering of states, evident as little squares of recurrence on the recurrence plot (Fig 4).

Before subjecting the data to RQA, each reading time series was logarithmized and z-scored to ensure that RQA would capture standardized sequential properties of the time series, which otherwise would have been conflated with differences in magnitude and variance between participants. Then, RPs were calculated using the delay parameter τ = 1, the embedding dimension parameter *D* = 5, Euclidean normalization of the phase-space, and a radius parameter *r* = 0.5. The parameters τ and *D* were determined following the guidelines by Wallot [60]. Each parameter was estimated individually for each participants’ time series, using the average mutual information procedure to estimate τ [61] and the false-nearest-neighbour algorithm to estimate *D* [62]. Then, the average (rounded to the nearest integer) values for τ and *D* were taken across all participants. The radius parameter *r* was set to yield at least a minimum of 1% recurrence, resulting in an average recurrence rate of 0.12 across all data sets. All RQA analyses were performed in Matlab, using the CRP-Toolbox for Matlab [63].

Next, the four recurrence measures—*%REC*, *%LAM*, *TT*, *maxV*–were subjected as dependent variables to three different linear mixed models implementing the three hypotheses outlined above. This was done using nonlinear spline models ([64], pp. 189–242) to estimate intercepts and slopes for the potentially different sub-phases in the reading time series (i.e., the random word reading task, the connected text reading task, the transition between the tasks). Model 1 implemented H1, assuming no transition between tasks and no differences in reading time dynamics between tasks, essentially implementing the hypothesis that the reading process is fully stable and similar across both tasks:

$$y_{ij}=\beta_{0i}+\beta_{1i}{Ti}_{ij}+\varepsilon_{ij},\;\varepsilon_{ij}∼N(0,\sigma_{\varepsilon }^{2})$$ (2)

Here, *y*_*ij*_ is the *i*^th^ participants value on an RQA-measures at time *j*, *β*_0*i*_ = *γ*_00_+*u*_0*j*_, *u*_0*j*_~*N*(0,*τ*_00_) is the individual intercept and *β*_1*i*_ is the fixed slope over times *Ti*_*ij*_. Model 2 implemented H2, assuming no substantial transition between the tasks, but incorporating a new level-parameter and slope for the second task, hence allowing for different reading time dynamics between random word and ordered text reading, essentially implementing the hypothesis that the reading process differs between tasks in level and slope, but no transition occuring:

$$y_{ij}=\beta_{0i}+\beta_{1i}{Ti}_{ij}+\beta_{2i}{Ph2On}_{ij}+\beta_{3i}{Ph2Ti}_{ij}+\varepsilon_{ij},\;\varepsilon_{ij}∼N(0,\sigma_{\varepsilon }^{2})$$ (3)

Here, *β*_2*i*_ is the fixed level-parameter for the onset of the second phase (i.e., the second reading task) *Ph*2*On*_*ij*_ and *β*_3*i*_ is the fixed slope over times *Ph2Ti*_*ij*_ for the second phase. Model 3 implemented H3, adding a third level-parameter and slope for a phase transition that might have occurred between the switching from one reading task to another and the settling of the reading process dynamics into the new task, essentially implementing the hypothesis that the reading process differs not only between the two reading tasks but also exhibits phase-transition-like behavior after the switch point:

$$y_{ij}=\beta_{0i}+\beta_{1i}{Ti}_{ij}+\beta_{2i}{TrOn}_{ij}+\beta_{3i}{TrTi}_{ij}+\beta_{4i}{Ph2On}_{ij}+\beta_{5i}{Ph2Ti}_{ij}+\varepsilon_{ij},\;\varepsilon_{ij}∼N(0,\sigma_{\varepsilon }^{2})$$ (4)

Here, *β*_4*i*_ is fixed level parameter for the onset of the phase-transition *TrOn*_*ij*_ and *β*_5*i*_ is the fixed slope over times *TrTi*_*ij*_ for the phase-transition. The problem with the new slope and level-parameters for the phase-transition in the third model was, that we did not know a priori the average duration of a phase-transition–if any–between tasks. Visual inspection of the RPs suggested that such a phase-transition could have spanned something between 100–300 words for the O→R condition (see Fig 4A), and something between 100–200 words for the R→O condition (see Fig 4B). In order to estimate the average length of the phase-transition region, we computed models with the intercept and slope for the phase-transition of lengths 100–300 and 100–200 and calculated Akaike’s Information Criterion (AIC) for each model. Lower values for AIC imply better model fit, and hence we selected the length of the phase-transition period (i.e., the number of data points that the intercept and slope parameters for the phase-transition were estimated for) based on the model with the smallest AIC value. The results were very similar across the four RQA measures, and based on this procedure the average length of the phase-transition was determined to be 190 for the O→R condition and 160 for the R→O condition (see the S1 Appendix to this paper for the resulting AIC plots).

Finally, in order to decide between the three hypotheses, we compared the three models implementing H1, H2 and H3 using pairwise χ^2^-tests to decide between the three hypotheses. Moreover, we tested the different parameters within the best-fitting model for significance in order to interpret the contribution of the different parameters to the model (i.e., whether the model indicated significant evidence for the difference in reading time dynamics between the different reading tasks and the occurrence of phase-transition-like behavior in reading times). The analyses were run separately for the O→R and R→O conditions in order to determine whether task order mattered for potential changes in reading time dynamics and task switching. All analyses were run in R (version 3.5.1) using the lme4 toolbox (version 1.1–17).

## Results

### Condition O→R

In the estimation procedure for the O→R condition, the average length of the phase-transition was determined to be 190 words. First, we present the results of χ^2^-tests that compare the three models implementing H1, H2 and H3 for each of the RQA measures—*%REC*, *%LAM*, *TT*, *maxV* (see Table 2). As can be seen, all comparisons for each of these four measures favour Model 3, implementing H3, the hypothesis that the time series of word reading times across random word list and connected text reading contains phase-transition-like behavior at the switch-point between the two reading tasks.

**Table 2:** Model comparison of H1, H2, and H3 for the four RQA measures for the O→R condition.

RQA measure	Model comparison	χ^2^	*df*	*p*	
*%REC*					
	H1 vs. H2	96.05	2	< .001	***
	H2 vs. H3	12.04	2	= .002	**
*%LAM*					
	H1 vs. H2	67.24	2	< .001	***
	H2 vs. H3	14.76	2	< .001	***
*TT*					
	H1 vs. H2	64.9	2	< .001	***
	H2 vs. H3	13.54	2	= .001	***
*maxV*					
	H1 vs. H2	62.59	2	< .001	***
	H2 vs. H3	10.23	2	= .006	**

Note.

* indicates *p* ≤ 0.05

** indicates *p* ≤ 0.01

*** indicates *p* ≤ 0.001.

As model three was selected throughout as the best model, we next test the parameters in model three for each of the four dependent measures. Regarding the evaluation of the phase-transition hypothesis, we see that the intercepts and slopes for the phase-transition region after the switch from random word list reading to ordered text reading are significantly different from zero for all four measures (Table 3). We did not know a priori whether the phase transition region would have been marked by a change in level or a change in the trend of recurrence measures–or both. However, we see that that the signs of the parameters are all negative, indicating indeed a loss of temporal structure during the phase transition.

**Table 3:** Parameter tests for within the models for each of the four RQA measures O→R condition.

RQA measure	Parameter	*B*	*SE*	*t*	*p*	
*%REC*						
	Intercept	0.048	0.027	1.80	= . 072	
	Slope_RandomWords_	0.004	0.000	7.49	< .001	***
	Level_Phase-Transition_	-0.161	0.023	-7.06	< .001	***
	Slope_Phase-Transition_	-0.004	0.001	-2.82	= .005	**
	Level_OrderedText_	0.085	0.025	3.45	< .001	***
	Slope_OrderedText_	0.001	0.002	0.72	= .469	
*%LAM*						
	Intercept	0.163	0.042	3.84	< .001	***
	Slope_RandomWords_	0.004	0.001	4.33	< .001	***
	Level_Phase-Transition_	-0.167	0.042	-3.94	< .001	***
	Slope_Phase-Transition_	-0.010	0.003	-2.91	= .004	**
	Level_OrderedText_	0.171	0.046	3.74	< .001	***
	Slope_OrderedText_	0.010	0.004	2.85	= .004	**
*TT*						
	Intercept	0.728	0.208	3.50	< .001	***
	Slope_RandomWords_	0.020	0.004	5.01	< .001	***
	Level_Phase-Transition_	-0.832	0.207	-4.01	< .001	***
	Slope_Phase-Transition_	-0.053	0.017	-3.14	= .002	**
	Level_OrderedText_	0.785	0.223	3.52	< .001	***
	Slope_OrderedText_	0.049	0.017	2.86	= .004	**
*maxV*						
	Intercept	1.111	0.385	2.89	= .004	**
	Slope_RandomWords_	0.037	0.008	4.84	< .001	***
	Level_Phase-Transition_	-1.564	0.385	-4.06	< .001	***
	Slope_Phase-Transition_	-0.087	0.031	-2.76	= .006	**
	Level_OrderedText_	1.246	0.414	3.01	= .003	**
	Slope_OrderedText_	0.083	0.032	2.58	= .010	**

Note.

* indicates *p* ≤ 0.05

** indicates *p* ≤ 0.01

*** indicates *p* ≤ 0.001.

Finally, we also see that the two tasks themselves appear to be more temporally structured compared to the phase-transition region, indicated by the positive signs of the parameters, and more over the significant positive slope indicate themselves that reading performance becomes increasingly structured with each task.

### Condition R→O

In the estimation procedure for the R→O condition, the average length of the phase-transition was determined to be 160 words. As in the analysis of the O→R condition above, we first present the results of model comparison and then the test of parameters of the selected models. As can be seen in Table 4, similar to the results found above, the Model 3 implementing H3 shows again consistently better fit compared the other two models. While Model 2 does not always show a significant improvement over Model 1, the model comparison again favors the model implementing H3 across all measures.

**Table 4:** Model comparison of H1, H2, and H3 for the four RQA measures for the R→O condition.

RQA measure	Model comparison	χ^2^	*df*	*p*	
*%REC*					
	H1 vs. H2	2.76	2	= .252	
	H2 vs. H3	27.24	2	< .001	***
*%LAM*					
	H1 vs. H2	9.77	2	= .008	**
	H2 vs. H3	10.09	2	= .006	**
*TT*					
	H1 vs. H2	8.37	2	= .015	*
	H2 vs. H3	9.11	2	= .010	**
*maxV*					
	H1 vs. H2	4.14	2	= .126	
	H2 vs. H3	14.97	2	< .001	***

Note.

* indicates *p* ≤ 0.05

** indicates *p* ≤ 0.01

*** indicates *p* ≤ 0.001.

Examining the parameter estimates sheds light on the different results pattern in the model comparison in the R→O condition compared to the O→R condition (Table 5). Even though Model 3 has been consistently selected in the model comparison, we do not see evidence for a prolonged phase-transition in the R→O condition. Across the four measures, all parameter estimates except one are not significantly different from zero, and the one effect that we observe on the slope parameter for the phase-transition on the *%REC* measure barely crossed significance. Still, all level parameters for the second task (here: connected text reading) are significant and the parameter estimates are positive, indicating an increase in the structure of reading times.

**Table 5:** Parameter tests for within the models for each of the four RQA measures R→O condition.

RQA measure	Parameter	*B*	*SE*	*t*	*p*	
*%REC*						
	Intercept	0.036	0.030	1.204	= .229	
	Slope_RandomWords_	0.004	0.001	5.963	< .001	***
	Level_Phase-Transition_	-0.030	0.033	-0.934	= .350	
	Slope_Phase-Transition_	-0.006	0.003	-1.987	= .047	*
	Level_OrderedText_	0.158	0.035	4.492	< .001	***
	Slope_OrderedText_	0.003	0.003	0.867	= .386	
*%LAM*						
	Intercept	0.139	0.035	3.930	< .001	***
	Slope_RandomWords_	0.006	0.001	5.912	< .001	***
	Level_Phase-Transition_	-0.071	0.051	-1.401	= .161	
	Slope_Phase-Transition_	-0.005	0.005	-0.939	= .348	
	Level_OrderedText_	0.120	0.055	2.192	= .028	*
	Slope_OrderedText_	-0.002	0.005	-0.327	= .744	
*TT*						
	Intercept	0.616	0.183	3.362	= .001	***
	Slope_RandomWords_	0.029	0.005	6.110	< .001	***
	Level_Phase-Transition_	-0.335	0.251	-1.333	= .183	
	Slope_Phase-Transition_	-0.029	0.025	-1.182	= .237	
	Level_OrderedText_	0.629	0.271	2.320	= .020	*
	Slope_OrderedText_	0.001	0.025	0.033	= .974	
*maxV*						
	Intercept	0.858	0.379	2.262	= .024	*
	Slope_RandomWords_	0.051	0.009	5.419	< .001	***
	Level_Phase-Transition_	-0.634	0.504	-1.258	= .208	
	Slope_Phase-Transition_	-0.041	0.050	-0.833	= .405	
	Level_OrderedText_	1.495	0.543	2.750	= .006	**
	Slope_OrderedText_	-0.015	0.050	-0.291	= .771	

Note.

* indicates *p* ≤ 0.05

** indicates *p* ≤ 0.01

*** indicates *p* ≤ 0.001.

## Discussion

The results from the O→R task switching condition are very much in line with what was expected from LGH. The findings provide us with a standard example of a phase-transition as a loss-of-temporal structure between the stable phases (i.e., the performances within each task). The positive slopes for the recurrence measures within each task suggests that there is still an increased adaptation of the cognitive processes with each reading condition, strengthening the notion that the reading process is not context-independent across different reading tasks but that reading reflects an interaction with the constraints of different reading tasks. Also, it is worth noting that the length of this transition spans–on average–about 190 words. That is, settling in from one reading task into another spans a range of the stimulus material that alone rivals or exceeds the size of text material that is currently used within the reading task to examine cognitive performance in that task.

The results R→O task switching are not wholly as expected. While the model that includes levels and slopes for the phase-transition regime has been chosen as the best fitting model, the slope parameters for the reading times are not significant themselves, even though their addition to the model improved the overall model fit, perhaps increasing the explanatory power of the levels and slopes of associated with each of the two reading tasks. This leaves open several interpretations. At first glance, the results do not seem to suggest evidence in favor of the phase-transition prediction. However, the model comparison suggests rather that perhaps the effect of the task transition changes with sequence, e.g., when going from random to connected text, reading exhibits less of the further increase in RQA measures that would have been expected under continued random word list reading. In any event, this sequence effect on the phase-transition seems more likely than the absence of a quick transition. After all, if there were no quick transition, Model 2 should not have performed worse than Model 3. Nevertheless, this is a speculative interpretation, and potentially a proper control condition is needed that contrasts the effects of task switching with continued task performance.

Taken together, results from the R→O and O→R condition could also be interpreted as a higher-order effect of a soft-assembling of the reading process, as they reveal what could be interpreted as a hysteresis effect. Broadly speaking, hysteresis is simply the dependence of the state of a system on its history. In our context, it means that the working of the cognitive processes involved in each of the two reading tasks depends on which of the two tasks came first. Similarly, Teng et al. [14] found that the presence of lexical effects in a lexical decision task dependent upon whether the lexical decision task was preceded by a story reading task or not. Even though this study did not investigate task switching, it aligns with our present findings by pointing to the fact that task order matters even for what have been thought to be fundamental processes of reading. As with Teng et al.’s findings, our present results show a higher-level nonlinearity that reveals itself in the order in which tasks are performed. At least such an interpretation would be consistent with LGH because it would also align with Wittgenstein’s [16] original suggestion, that there are not only different language games with different “rules” (i.e., regularities according to which language is processed and is meaningfully interpreted), but also different families of language games that are more or less similar to each other. Perhaps we are seeing a difference due to familiarity with ordered text that draws a reader in to the task and to the relative lack of engagement with a random-word list. Readers may feel themselves more closely related to those families of readings games that more closely resemble reading in more natural, everyday settings.

This issue of familiarity and the background experience of the reader led us to consider new hypotheses that warranted further study. One hypothesis is that the effect pattern observed here emerges though an interplay of reader skill and absence vs. presence of meaning in the different tasks: For instance, readers might transition more easily and quickly from random word reading into connected text reading which is the more stable performance state. Moreover, the inconclusive results pattern for the R→O condition might furthermore be a function of reading skill and familiarity with a language. Native speakers of English may manage such a transition too quick to be observable in our measures, but a group of less-skilled readers not as familiar with that language might exhibit such a transition effect also in the R→O condition. Hence, a second study was conducted to test these ad-hoc hypotheses.

## Study 2

The aim of study 2 was to investigate whether a more consistent pattern for phase-transitions in reading times could be observed with a sample of less-skilled readers who are less familiar with the language of the text. Our remarks preceding Study 1 and the results following from Study highlighted long-unaddressed context-sensitivity of reading. That is, reading is a complex process whose dynamics might be rooted in single-word recognition but quickly bleed from single-word levels to discourse processing at the sentence- or paragraph-level and again to the whole-text narrative level. All of that context sensitivity manifest in strictly monolingual readers. Second-language (L2) reading embodies a dramatic accentuation of that context sensitivity offering an excellent test case to compare with Study 1. Not only does L2 reading reflect all of the same interactions across level, from word to discourse and narrative contexts, within the L2 text, but L2 readers’ ability to navigate an L2 text depends further on their own individual differences in command with their first language (L1) [65–69].To that end, Danish native speakers who spoke English as their second language (L2) were recruited to perform the same reading tasks as in Study 1, using English texts. Testing second-language bilinguals potentially provides evidence for the generality of the phase-transition effect across languages and native and non-native speakers. Moreover, we collected subjective ratings of text meaningfulness in order to investigate the effect of the task manipulation on subjective meaning. We suspect that the ability of participants to extract or construct meaning from randomized words depends on their prior reading experience (i.e. the presence vs. absence of an intact text), and that this in turn might modulate aspects of the phase-transition-like behavior during task switching.

## Materials and methods

### Participants

Thirty participants, undergraduate students of Aarhus University, Denmark (average age = 20.90, *SD* = 1.52, ranging from 19 to 24 yrs.; 17 were female) participated in the study. The words and the text were presented in English, and all participants were native speakers of Danish (L1) and had English as their second language (L2). All of them had normal or corrected-to-normal vision.

### Ethics approval

The study was reviewed and approved by the Scientific Committees for Region Midtjylland (Danmark).

### Materials

As in study 1.

### Procedure

As in study 1, except that participants were asked how meaningful they found the first and second half of presented text to be, rated on a 7pt. scale from “not at all meaningful” to “very meaningful” after they finished reading.

### Data analysis

**Fig 5:**
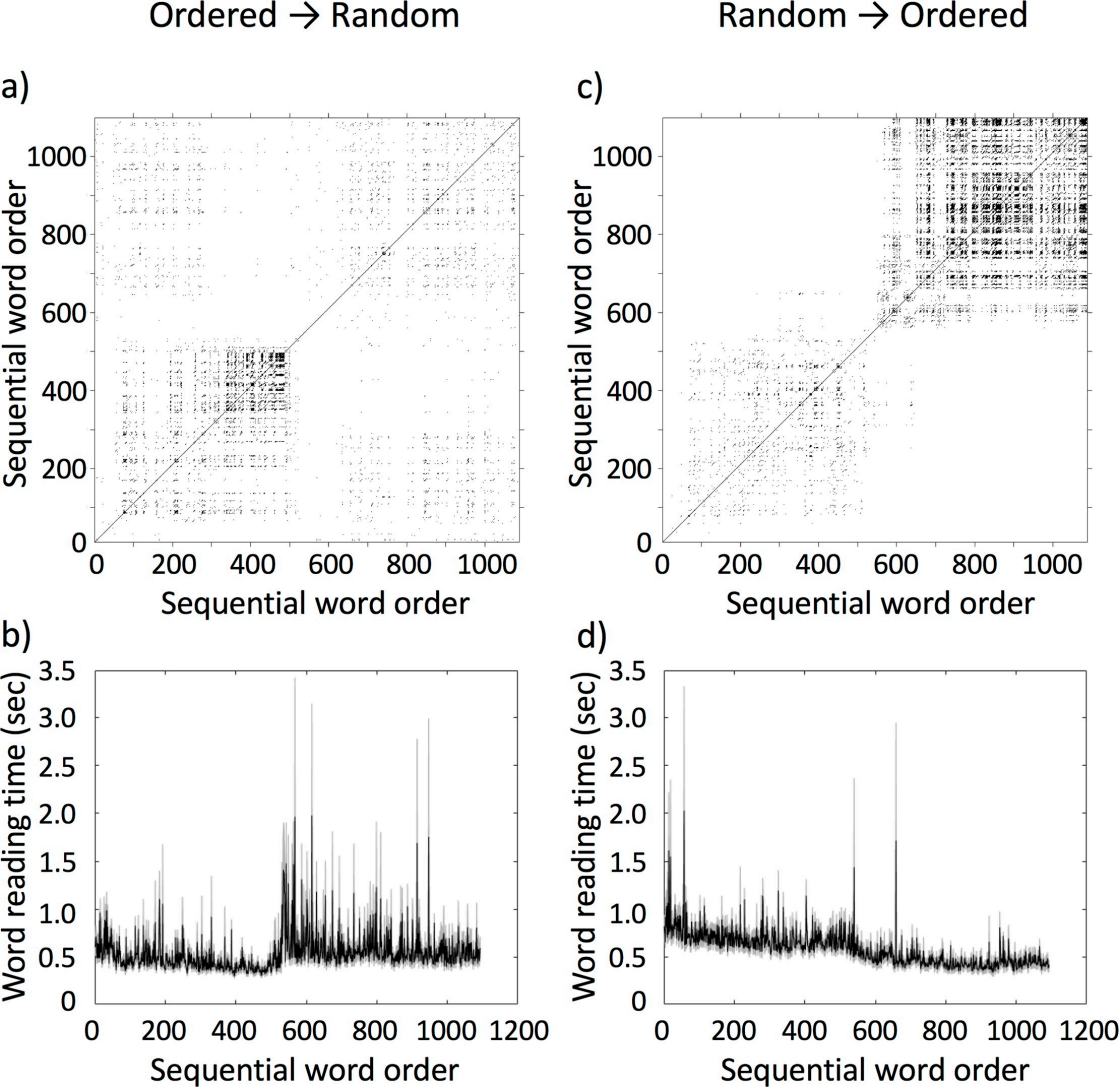
Example recurrence plots and word reading series. Average RP of the O→R condition (a) and the associated average time-series of word reading times (averages in black and standard deviations in grey) (b), and average RP of the R→O condition (c) and the associated average time-series of word reading times (averages in black and standard deviations in grey) (d) for Study 2.

As in study 1, except that we needed to estimate again the length of a potential phase-transition for the respective parameters in the model. Again, visually inspecting the RPs suggested that such a phase-transition could have spanned something between 50–200 words for both, the O→R and the R→O condition (see Fig 5A and 5B). Here, we continued with the same procedure outlined in Study 1 to estimate the average length of the phase-transition region using the calculation of AIC for each model (see the S2 Appendix to this paper for the resulting AIC plots). In order to decide between the three hypotheses, again proceeded in the same way as in Study 1, first comparing the three models implementing H1, H2 and H3 using pairwise χ^2^-tests and then testing the different parameters within best-fitting model for significance. Again, analyses were run separately for the O→R and R→O conditions. Meaningfulness ratings were analyzed using a simple 2x2 mixed repeated measures ANOVA with the between-participant factor Order (2 levels: R→O vs O→R) and the within-participant factor tasks (2 levels: random word list reading vs. connected text reading).

## Results

### Condition O→R

In the estimation procedure for the O→R condition, the average length of the phase-transition was determined to be 50 words. Just as in study 1, Model 3 implementing H3 was consistently selected as the most informative model in the model comparison test (see Table 6). In contrast to the O→R condition in study 1, when testing for the individual model parameters, we do see comparatively weaker effects of levels or intercepts for the phase-transition (and no effect for the *maxV* measure). Still, as the signs of the coefficients are generally negative, the measures indicate that the transition after the switch-point induced a loss in reading-time structure, either in level or development (see Table 7).

**Table 6:** Model comparison of H1, H2, and H3 for the four RQA measures for the O→R condition.

RQA measure	Model comparison	χ^2^	*df*	*p*	
*%REC*					
	H1 vs. H2	101.67	2	< .001	***
	H2 vs. H3	17.95	2	< .001	***
*%LAM*					
	H1 vs. H2	71.49	2	< .001	***
	H2 vs. H3	31.51	2	< .001	***
*TT*					
	H1 vs. H2	78.19	2	< .001	***
	H2 vs. H3	32.44	2	< .001	***
*maxV*					
	H1 vs. H2	90.13	2	< .001	***
	H2 vs. H3	26.45	2	< .001	***

Note.

* indicates *p* ≤ 0.05

** indicates *p* ≤ 0.01

*** indicates *p* ≤ 0.001.

**Table 7:** Parameter tests for within the models for each of the four RQA measures O→R condition.

RQA measure	Parameter	*B*	*SE*	*t*	*p*	
*%REC*						
	Intercept	0.051	0.024	2.147	= .032	*
	Slope_RandomWords_	0.003	0.000	7.107	< .001	***
	Level_Phase-Transition_	-0.054	0.025	-2.171	= .030	*
	Slope_Phase-Transition_	-0.009	0.005	-1.604	= .109	
	Level_OrderedText_	-0.037	0.029	-1.276	= .202	
	Slope_OrderedText_	0.007	0.005	1.269	= .205	
*%LAM*						
	Intercept	0.136	0.044	3.116	= .002	**
	Slope_RandomWords_	0.004	0.001	6.252	< .001	***
	Level_Phase-Transition_	0.008	0.057	0.138	= .890	
	Slope_Phase-Transition_	-0.047	0.022	-2.162	= .031	*
	Level_OrderedText_	-0.061	0.074	-0.822	= .411	
	Slope_OrderedText_	0.044	0.022	2.036	= .042	*
*TT*						
	Intercept	0.656	0.198	3.314	= .001	***
	Slope_RandomWords_	0.022	0.003	6.433	< .001	***
	Level_Phase-Transition_	-0.028	0.266	-0.107	= .915	
	Slope_Phase-Transition_	-0.216	0.102	-2.128	= .033	*
	Level_OrderedText_	-0.310	0.348	-0.890	= .374	
	Slope_OrderedText_	0.205	0.102	2.014	= .044	*
*maxV*						
	Intercept	0.947	0.391	2.421	= .015	*
	Slope_RandomWords_	0.044	0.006	7.062	< .001	***
	Level_Phase-Transition_	-0.554	0.498	-1.113	= .266	
	Slope_Phase-Transition_	-0.244	0.190	-1.284	= .199	
	Level_OrderedText_	-0.978	0.651	-1.502	= .133	
	Slope_OrderedText_	0.215	0.190	1.129	= .259	

Note.

* indicates *p* ≤ 0.05

** indicates *p* ≤ 0.01

*** indicates *p* ≤ 0.001.

### Condition R→O

In the estimation procedure for the R→O condition, the average length of the phase-transition was determined to be 120 words. Again, Model 3 implementing H3 was consistently selected as the best fitting model in the model comparison test (see Table 8). Contrary to what we observed in the R→O condition in Study 1 investigating transitions between reading tasks in native speakers, the parameters for the phase-transition levels and slopes are generally significant (except for *%REC*) and indicate an increase in the level or slope temporal structure in reading times, except for the negative level parameter for *%LAM* (see Table 9).

**Table 8:** Model comparison of H1, H2, and H3 for the four RQA measures for the R→O condition.

RQA measure	Model comparison	χ^2^	*df*	*p*	
*%REC*					
	H1 vs. H2	8.53	2	= .014	*
	H2 vs. H3	22.81	2	< .001	***
*%LAM*					
	H1 vs. H2	12.33	2	= .002	**
	H2 vs. H3	14.43	2	< .001	***
*TT*					
	H1 vs. H2	13.47	2	= .001	***
	H2 vs. H3	13.76	2	= .001	***
*maxV*					
	H1 vs. H2	12.01	2	= .002	**
	H2 vs. H3	21.10	2	< .001	***

Note.

* indicates *p* ≤ 0.05

** indicates *p* ≤ 0.01

*** indicates *p* ≤ 0.001.

**Table 9:** Parameter tests for within the models for each of the four RQA measures R→O condition.

RQA measure	Parameter	*B*	*SE*	*t*	*p*	
*%REC*						
	Intercept	0.072	0.022	3.300	= .001	***
	Slope_RandomWords_	0.000	0.000	0.792	= .428	
	Level_Phase-Transition_	-0.043	0.029	-1.471	= .141	
	Slope_Phase-Transition_	0.006	0.007	0.865	= .387	
	Level_OrderedText_	0.057	0.035	1.628	= .103	
	Slope_OrderedText_	-0.008	0.007	-1.104	= .270	
*%LAM*						
	Intercept	0.160	0.039	4.062	< .001	***
	Slope_RandomWords_	0.001	0.001	1.844	= .065	
	Level_Phase-Transition_	-0.102	0.047	-2.145	= .032	*
	Slope_Phase-Transition_	0.024	0.007	3.349	= .001	***
	Level_OrderedText_	-0.155	0.053	-2.932	= .003	**
	Slope_OrderedText_	-0.027	0.007	-3.807	< .001	***
*TT*						
	Intercept	0.862	0.196	4.409	< .001	***
	Slope_RandomWords_	0.005	0.004	1.211	= .226	
	Level_Phase-Transition_	-0.371	0.223	-1.665	= .096	
	Slope_Phase-Transition_	0.097	0.030	3.199	= .001	***
	Level_OrderedText_	-0.644	0.245	-2.625	= .009	**
	Slope_OrderedText_	-0.113	0.030	-3.711	< .001	***
*maxV*						
	Intercept	1.322	0.349	3.789	= .001	***
	Slope_RandomWords_	0.005	0.007	0.707	= .480	
	Level_Phase-Transition_	-0.784	0.409	-1.917	= .055	
	Slope_Phase-Transition_	0.236	0.056	4.217	< .001	***
	Level_OrderedText_	-1.604	0.450	-3.562	< .001	***
	Slope_OrderedText_	-0.257	0.056	-4.605	< .001	***

Note.

* indicates *p* ≤ 0.05

** indicates *p* ≤ 0.01

*** indicates *p* ≤ 0.001.

### Meaning ratings by task and condition

**Fig 6:**
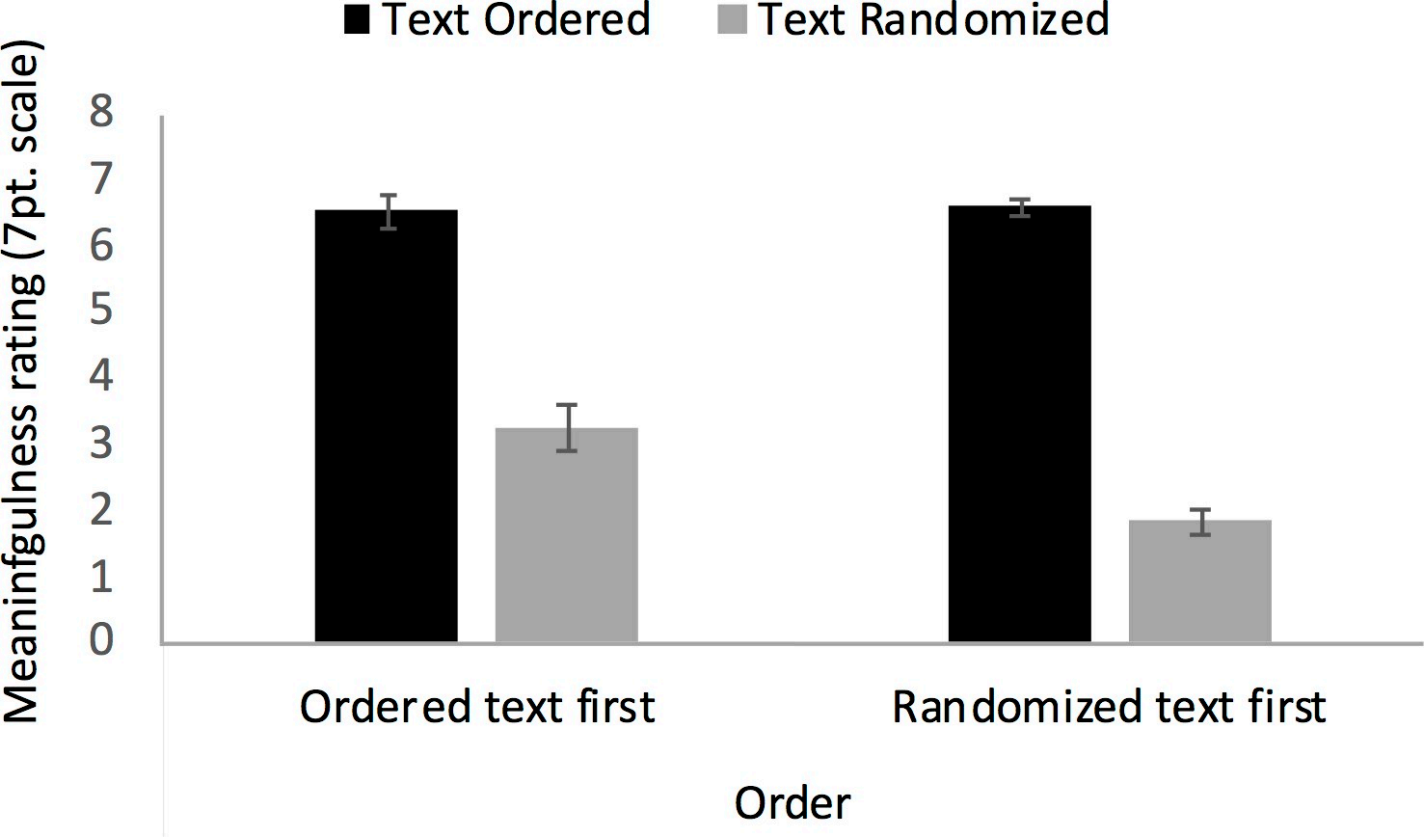
Meaningfulness ratings. Average meaningfulness ratings by reading tasks (random word list reading vs. connected text reading) and order (O→R vs. R→O). The error bars indicate the standard error of the mean.

Meaningfulness ratings were analyzed using a simple 2x2 repeated measures ANOVA with the between-groups factor Order (2 levels: R→O vs O→R) and the within-participant factor Task (2 levels: random word list reading vs. connected-text reading). There were significant main effects of Order (*F*(1, 28) = 6.66, *p* = .001) and Task (*F*(1, 28) = 177.15, *p* < .001), as well as an interaction between the factors Order x Task (*F*(1, 28) = 5.95, *p* = .021). As can be seen in Fig 6, this pattern of effects was a consequence of average meaningfulness ratings being of similar (and near ceiling) magnitude in the connected text reading condition and generally lower in the random word list reading condition, with the difference that meaningfulness ratings were higher when random word list reading was the later rather than the earlier of the two tasks. Hence, experience with the intact text allowed participants to see some bits and pieces of a meaningful story throughout the randomized words, when they had been previously exposed to that story.

## Discussion

As in Study 1, we tested the hypothesis that switching between two reading tasks leads to phase-transition like behavior in reading times also in L2 readers. Moreover, we speculated to find a more consistent pattern of phase-transitions for both task orders, O→R and R→O, insofar as the presence of such a transition phase was influenced by the lower familiarity of L2 readers compared to the L1 readers. Our analysis shows that Model 3 implementing H3 was consistently selected as the best model throughout in Study 2, indicating that the addition of intercepts and slopes for the phase-transition regime improved model fit. For the O→R condition, we did find similar effects as in study 1, but contrary to our expectations, these effects were attenuated, not pronounced. However, we did now find effects of the phase-transition parameters in the R→O condition, but they were not consistently marked by negative signs, indicating a loss of structure, but also showed increased gain in temporal structure, albeit from a lowered level. So, in line with our expectations, some of the parameters for the phase-transition regime were now found to be significantly different from zero in the reading times of the L2 sample compared to the L1 sample in Study 1. However, also study 2 shows that the transitions from O→R compared to R→O are indeed asymmetrical.

The meaningfulness ratings collected provide a hint to some of the causes of this asymmetric transition: while all participants gave generally high meaningfulness ratings to the connected text reading task and low meaningfulness ratings to the random-word list reading task, participants still provided higher ratings of meaningfulness to random-word list reading when the connected text reading task came first as opposed to when the connected test reading task came second. When participants started with the random word list reading task, they may have perceived the word they read as utterly meaningless, but readily adapted to connected text reading where a coherent story was presented. However, when participants started with the connected text reading task and switched to the random word list reading task, they seem to cognitively hang on to the story of the connected text reading task, trying to extract meaning in line with that story during the following random word list reading, even though this was obviously difficult to impossible. Nevertheless, it was probably hard for them to give up searching for such meaning, which might be the proximal cause of the extended phase-transition period that we observed in O→R condition in study 2.

## General discussion

In the current study, we tested two hypotheses about what happens at the switch-point between two different reading tasks. The first prediction was inferred mainly from the current practice of conducting experiments in reading research but also accompanying theory. This led us to suggest that the reading process–or more specifically, its different components–are thought to be relatively task independent and quick to adapt to new reading tasks, akin to a hard-wired processing architecture that will readily process any input that is encountered. In contrast, we derived a second prediction about what happens at the switch-point between two reading tasks from the language game hypothesis (LGH) of reading. Here, the reasoning is that the cognitive processes involved in reading are not hard-wired, but soft-assembled. That is, they are not constantly available in the background of the cognitive architecture, but emerge as an interplay between organism and (reading) task. Moreover, the hypothesis was motivated by problems from the philosophy of language, particularly the problem of how meaning in language can be grounded and be flexibly constructed. Here, we interpreted reading in terms of Wittgenstein’s [16] concept of language game, where a particular context of language use (here: reading) is not just dependent on an individual’s language capacities and the structure of a given language, but is inherently context dependent. Drawing from LGH, we predicted that the switch from one reading task to another entails a change in the way language is processed and a re-assembling of the cognitive architecture in accordance with the specific constraint of each task. Hence, we predicted the occurrence of phase-transition like behavior in reading times when switching from one task to another.

Examining the recurrence properties of reading times, we found evidence for such phase-transitions in native speakers (L1) and second language readers (L2) when switching from connected text reading to random word reading. However, we did not find clear evidence for native speakers when switching from random word reading to connected text reading with regard to the phase-transition parameters. These findings paint a rather mixed picture, because models that contained predictors for the transition phase consistently exhibited better fit according to AIC compared to models that did not include parameters for the transition phase. The problem here was that the estimates associated with the phase-transition were not significantly different from zero for the native speakers that switched from random word list reading to connected text reading.

From the perspective of LGH, which is primarily based on the concept of the language game, this pattern of effects was only partially expected, because the assumption was that phase-transitions between reading tasks should occur irrespectively of task order. From the underlying assumption of self-organization of the cognitive system, such asymmetric patterns, called hysteresis, are expected, however, and have been observed in other domains of cognitive performance, for example visual perception [70], motor coordination [71], or speech perception [72]. Hence, with regard to the different hypotheses, the results are fully in line with a self-organized architecture of the cognitive system, partially support LGH, but do not provide evidence for the hypothesis that the reading process is hard-wired, sufficiently context-independent across different reading tasks, nor for the assumption that the cognitive system is ready to process linguistic input stably at a short onset period.

Study 2 provided additional information as to why hysteresis effects between the two orderings of tasks occurred. First of all, we collected meaningfulness ratings from participants, which showed that the connected text is relatively invariably judged to be meaningful at ceiling, while the perceived meaningfulness of the random text reading task depends on whether the connected text reading task came first or second: When participants started with random text reading, the text was judged to be near fully meaningless, but when participants started with the connected text reading task, the random word reading task was judged to be somewhat more meaningful. It seems that when participants have already extracted overarching meaning of a text, such meaning continues to “shine through” even when the constituent words become randomized. This might increase the difficulty to settle into the new task, because participants have problems to disengage from the first task and continue to try for a longer time to find meaningful relations that they know–or suspect–to be there from the sequence of randomized words, while the same sequence is found to be simply meaningless if prior experience does not suggest otherwise. This interpretation is in line with observations that bilinguals have repeatedly shown smaller switch costs than monolinguals [73,74]. It remains controversial as to whether this benefit reflects a specific linguistic mechanism or general executive control [75], but clarifying this latter question does not is immaterial to the fact that we find bilinguals do persist in showing lower switch costs. What replicating this effect here suggests is that experience with bilingualism might possibly indicate that bilinguals experience flexibility even after having made use of longer-scale structure as in discourse to connect singly presented words.

Moreover, Study 2 found weaker effects of some of the phase-transition parameters in the O→R condition, but with the expected negative signs for loss of structure in reading times during a phase-transition in L2 readers. However, study 2 found also significant effects for the phase-transition parameters for the R→O condition, which suggest a steeper increase in reading time structure at the transition from random word to ordered text reading. Even though we expected a uniform pattern of phase-transitions across studies and conditions, this effect might suggest that the ordered text reading task works as a stronger cognitive attractor, which does not so much pertrub, but stabilize reading performance. That we did not find such an effect for L1 reader we suspect is due to the lower familiarity and hence lower reading skill with an acquired language compared to a native language. While L1 readers perhaps mastered this transition smoothly, this was not the case for L2 readers. Assuming that it is easier to read an ordered text compared to a list of random words over a longer period of time, the increase in reading time structure in L2 readers might indicate that the ordered text supported their reading process, while this was not equally necessarily for the more skilled L1 readers. Staying with the terminology of dynamics systems, lower reading skill acts change the attractors strengths of different reading task. That the ordered text reading task provided the stronger attractor was also supported by pattern of meaningfulness ratings collected in study 2.

The weaker effects of the phase-transition parameters in the O→R condition might have their roots in higher inter-individual variability of the L2 readers recognizing the swich in tasks. However, sample size limitations did not allow us to add participant slopes to our models, which resulted in convergence problems. That this might however be the reason is also suggested by qualitative statements of some participants who reported after their participation in the experiment that they initially did not recognize that words started to appear in an ordered sentence structure, and that it took them a while (and some time continuing reading) to fully realize this. However, we did not systematically ask participants to indicate whether this was a common experience in our sample or not.

Still, this overall suggests that the perspective that different reading tasks constitute different attractor-like state of the cognitive systems, and that specifically random word reading is a weaker cognitive (attractor) state compared to connected text reading, as reading settles more strongly and quickly from random word to connected text reading, while the connected text reading task seems let readers transition much more slowly to another task. Our results suggest that relevant factors for the constitution of the different task performances are the presence of meaning (and hence the presence of underlying linguistic structures which allow a sequence of words to be understood as meaningful text compared to a mere collection of individual words), which governs the relative strength of the different types of cognitive organizations between the tasks, and furthermore reading skill, which also governs the ease-of-transition between those tasks. Moreover, we observed these effects not only in native speakers (L1), but also second language readers (L2), which provides initial evidence for the putative universality of this pattern across languages.

Finally, our observations on reading also have implications that potentially go beyond the fields of reading research and language comprehension. Specifically, there is a broad literature of task switching as a means to investigate properties of attention and cognitive resource taxation. The language game hypothesis offers a timely resolution to longstanding questions in the task-switching literature. Switching from one task to another often exhibited a brief burst in response time, suggesting increased processing cost. However, the literature long respected two competing sources of this cost: reconfiguration or interference. That is, either we face the challenge of redirecting our executive functions or the challenge of forgetting old associations with the same or similar stimuli. The former source of costs is a top-down reconfiguration of the cognitive system as it identifies and settles into a new task set, and the latter source of costs is a bottom-up interference of stimulus features with past stimulus-task associations [76]. The reconfiguration account suited a memoryless transition that had only to do with higher-order expectations and task-set parameters, and in particular, it suited any evidence of a switch cost that did not depend on the length or stimulus details of the previous task. However, the interference account aimed to explain a growing set of findings that suggested that stimulus, task, and response actions did have longer-range entailments across the switch in task [77]. Making matters more challenging were findings that reconfiguration and interference were not mutually exclusive opposites, but rather different ways to invoke interactions across various levels in a hierarchically organized cognitive system—all of which could constitute cost with task switching [78,79]. Here we see the interactions across scale that the language-game hypothesis is ready to anticipate.

More recent research has only served to accentuate the turbulence of hierarchically spreading interactions through task-switching. Subsequent research has reasserted this hierarchical knittings-together of top-down and bottom-up control [80,81]. Furthermore, elaborations of designs and modeling strategy have furrowed the notion of switching costs into ever finer scales of time and rooted them in finer subdivisions of the cognitive system. For instance, as experimenters have tested task switching not just across block but also within block, they need to distinguish between global and local costs of switching, respectively [82] or, by another-yet-synonymous set of labels, switching costs and mixing costs, respectively [83]. Local/mixing costs may reflect that trial-by-trial variation in the task set never permits full reconfiguring the task set, and more global/switching costs may come from maintaining multiple tasks sets [82], but the costs of having to inhibit interfering task-stimulus relationships seem to accrue across time, no matter their source [84].

The task-switching literature has indeed addressed asymmetric switching costs such as we find. Switching costs have seemed to be greater when switching from more difficult tasks to easier tasks, rather than from easier to more difficult tasks [75,85]. Meuter and Allport [85] acknowledged that this asymmetry could well seem paradoxical outside of the appropriate theoretical perspective. Specifically, they raised this point in their study of bilinguals engaging in task switching from L1 to L2 or from L2 to L1, which found the greater switching costs in the latter case. Their explanation of this asymmetric switching cost had more to do with the heavy cost of inhibiting the more familiar L1. Inhibiting a more familiar language across the entire first block of the study proved a larger investment of cognitive resources and required a longer, slower reconfiguration than making the first L2 response after a block spent not inhibiting L1. The full extent of this investment of cognitive resources that Meuter and Allport imagine is really stunning: the asymmetric switch cost from L2 to L1 was so insensitive to the details of individually presented stimuli that they were convinced that reconfiguration and inhibition operated upon the whole-language lexicon and did not have some graded investment according to task particulars.

The present findings manage to fall beyond the predictions of task-switching literature without actually conflicting with any of the theoretical perspectives. LGH welcomes the above-mentioned contribution of participant-dependent constraints impinging on task performance, but besides sharing the reluctance to commit to effects of task-specific difficulty/easiness, we are unsure how to identify the “difficult” versus “easy” task amongst our options. If we called our participants “bilingual” in their capacity to process text in two different formats, i.e., to read narratively-ordered text (a more natural “L1”) as well as to read randomly-ordered text (a more contrived “L2”), then the longer transition time for R→O would suggest the opposite results from the task-switching literature [75,85]. If we had built in more explicit transition and provided participants with some longer cue-to-target interval to plan a new response, we might have allowed some better preparation, but the effect of such a manipulation would have depended on our participants being actually bilingual—and not in the sense of being able to read words in the same language with or without narrative order, which sense surely describes the monolinguals who did not respond to longer cue-to-target intervals [83].

What we have documented here is that, when we allow the monolinguals to play different language games within the same language, we can generate asymmetric switch costs among monolinguals. We do not need multiple languages, and we do not even need to ask for different responses from block to block. We need only to present single words at a time, with or without dependence across trials. The recurrent structure across button presses indicates that each single response reflects a longer-scale appreciation of longer-scale relationship across the different words. And having allowed participants to wind a longer-scale relationship around these individually presented words, they have invoked their own inertia and, in effect, fabricate their own switch costs.

Longer-scale structure in our task makes us skeptical about boiling the current asymmetry in switching costs down to known mechanisms in the task-switching literature. More crucially, better comparison would require our tasks affording only local response to local stimulus features. To our knowledge, there is no analogue in the task-switching literature that allows participants to respect any linkage of the individual stimuli across trials. What the task-switching literature provides includes linguistic task-switches from one language to another or with non-linguistic task-switches from judging colors to judging shapes (or vice versa) for the same stimuli. Much though the task-switching literature has come to appreciate the accumulations across time and interactions across levels of processing [80–83], task-switching research has yet to give participants a task that asks them to embody precisely this interactive accumulation.

Here we have used the same stimuli, the same responses, and the same language across tasks. We have thus generated an asymmetry in switching costs where task-switching literature suggests no likely explanation. Our findings may even vindicate the colorful words of Wittgenstein about the internal complexity of just a single language: ‘Our language can be seen as an ancient city: a maze of little streets and squares, of old and new houses, and of houses with additions from various periods; and this surrounded by a multitude of new boroughs with straight regular streets and uniform houses.’ Naturally, the task-switching literature’s strict adherence to tasks independence of stimuli across tasks certainly makes findings of interactive accumulation all the more striking, but forcing the independence of stimuli across trial risks underestimating the actual switch costs on two counts: first, by refusing participants more room to integrate their task experience and second, by minimizing the number of time points and time scales at which we test for these costs. The present results suggest that participants are integrating much more than we instruct them to, definitely in our task but also possibly in the standard task-switching studies as well.

In any case, in many of the paradigms summarized above, switching from task to task (e.g., from number to letter, langauge to language) is expected to occur very quickly, but our findings suggest that even for such highly trained performances such as reading, there are transition periods that endure across a range of 50–190 words on average before the participating cognitive processes have actually settled stably into regular task performance. This means that the observed performance when switching from one task to another on the bases of single (of a few) trials might really only tap into a perturbated kind of performance or a transitory performance that is not like the actual task performance that one seeks to investigate. Hence, our results suggest that task switching has to be observed across multiple trials or a more extended period of time where participants get a chance to perform consecutively longer within each task, and that the performance observed at the onset of a new task (i.e., the phase-transition period) contains potentially very valuable information about shifts in attention, allocation of cognitive resources and re-organization of cognitive processes associated with such a switch in tasks.

### Limitations and outlook

The current study, particularly with its application in reading, has several limitations that are methodological in nature. First of all, the data here were collected using a self-paced reading paradigm, and while self-paced reading exhibits many of the same effects that can be observed using, for example, eye tracking [86], our observations are limited to a sampling on a word-by-word basis, and more high-frequent measurements (such as eye movement or EEG recordings) might provide a clearer picture of what happens at the onset of task switching. This coarse grain of the measure may explain why we did not find a clear pattern of effect when switching from random word to connected text reading.

Moreover, the notion of soft-assembly and self-organization of cognitive processes in the interaction with a particular task was measured on a very abstract level, namely through the occurrence of fluctuation patterns that resemble nonlinear phase-transitions. While this is a valid prediction given self-organization of the cognitive architecture, we have neither shown a qualitative change in specific cognitive processes, nor have we shown how the two reading tasks night differ with regard to language processing. The former would require a multivariate measurement of the different cognitive processes that are integratively involved in reading, which could perhaps be obtained via EEG or time-dependent fMRI measures, while the latter would require to show how particular kinds of (linguistic) information in the text drives those cognitive processes differently in each of the two reading tasks. Obviously, such analysis requires multivariate measurements that are not available using self-paced reading. Finally, a particular limitation to the analysis of study 2 is the lack of information about the degree of language skills participants have in English as their second language. Unfortunaltey, no such information has been obtained from participants.

Looking forward from the present studies, future research does not only need to address these methodological limitations, but also provide a more stringent theoretical understanding of the nature of meaning and its role in the different reading tasks. Furthermore, a practical goal of the current research paradigm could be to chart the “family relations” between different reading tasks that are currently used in research on reading. This would allow to group tasks for which more specific processing models of written language perception can be formulated, and it would furthermore allow to define the boundary conditions for which one would expect these models to hold.

## Supporting information

S1 File AIC plots study 1. (DOCX)

S2 File AIC plots study 2. (DOCX)

S3 File Description of data file. (DOCX)

S4 File Data file. (CSV)

## References

[1] Clifton C Jr., Duffy SA. Sentence and text comprehension: Roles of linguisticstructure. Ann Rev Psychol. 2001;52:167–196. 11148303 10.1146/annurev.psych.52.1.167

[2] Graesser AC, Millis KK, Zwaan RA. Discourse comprehension. Ann Rev Psychol. 1997;48:163–189. 15012477 10.1146/annurev.psych.48.1.163

[3] Rayner K. Eye movements in reading and information processing: 20 years of research. Psychol Bull. 1998;124:372–422. 9849112 10.1037/0033-2909.124.3.372

[4] McNamara DS, Magliano JP. Towards a comprehensive model of comprehension In: Ross BH, editor. The psychology of learning and motivation. New York: Academic Press; 2009, pp. 297–384.

[5] Engbert R, Nuthmann A, Richter EM, Kliegl R. SWIFT: A dynamical model of saccade generation during reading. Psychol Rev. 2005;112:777–813. 10.1037/0033-295X.112.4.777 16262468

[6] Reichle ED, Rayner K, Pollatsek A. Eye movement control in reading: Accounting for initial fixation locations and refixations within the E-Z Reader model. Vis Res. 1999;39:4403–4411. 10789433 10.1016/s0042-6989(99)00152-2

[7] Reichle ED, Warren T, McConnell K. Using EZ Reader to model the effects of higher level language processing on eye movements during reading. Psychonom Bull Rev. 2009;16:1–21. 10.3758/PBR.16.1.1 PMC2629133 19145006

[8] McNerney MW, Goodwin KA, Radvansky GA. A novel study: A situation model of analysis of reading times. Discourse Processes. 2011;48:453–474.

[9] Menninghaus W, Wallot S. Eye movements reflect aesthetic reward during poetry reading. Manuscript submitted for publication. 2018;

[10] Richter T. What is wrong with ANOVA and multiple regression? Analyzing sentence reading times with hierarchical linear models. Discourse Processes. 2006;41:221–250.

[11] Schroeder S. What readers have and do: Effects of students' verbal ability and reading time components on comprehension with and without text availability. J Educational Psychol. 2011;103:877–896.

[12] Zwaan RA, Magliano JP, Graesser AC. Dimensions of situation model construction in narrative comprehension. J Exp Psychol Learning Mem Cognit. 1995;21:386–397.

[13] Wallot S, Hollis G, van Rooij M. Connected text reading and differences in text reading fluency in adult readers. PLoS ONE. 2013;8:e71914 10.1371/journal.pone.0071914 23977177 PMC3748108

[14] Teng D, Wallot S, Kelty-Stephen DG. Single-word recognition need not depend on single-word features: Narrative coherence counteracts effects of single-word features that lexical decision emphasizes. J Psycholing Res. 2016;45:1451–1472. 10.1007/s10936-016-9416-4 26861216

[15] Wallot S. From ‘cracking the orthographic code’ to ‘playing with language’: Toward a usage-based foundation of the reading process. Front Psychol. 2014;5:891 10.3389/fpsyg.2014.00891 25202285 PMC4141234

[16] Wittgenstein L. Philosophical investigations Hoboken, NJ: Wiley-Blackwell; 1953/2010 pp. 1–592.

[17] Wallot S. Understanding reading as a form of language-use: a language game hypothesis. New Ideas Psychol. 2016;42:21–28.

[18] Frost R. A universal approach to modeling visual word recognition and reading: Not only possible, but also inevitable. Behav Brain Sci. 2012;35:310–329. 23251930 10.1017/s0140525x12000635 PMC3662963

[19] Kello CT, Brown GD, Ferrer-i-Cancho R, Holden JG, Linkenkaer-Hansen K, Rhodes T, et al Scaling laws in cognitive sciences. Trends Cognit Sci. 2010;14:223–232. 20363176 10.1016/j.tics.2010.02.005

[20] Van Orden G, Holden JG, Turvey MT. Self-organization of cognitive performance. J Exp Psychol Gen. 2003;132:331–350. 10.1037/0096-3445.132.3.331 13678372

[21] Holden JG, Choi I, Amazeen PG, Van Orden G. Fractal 1/ƒ dynamics suggest entanglement of measurement and human performance. J Exp Psychol Hum Percept Perf. 2011;37:935–948. 10.1037/a0020991 21133553

[22] Turvey MT. Action and perception at the level of synergies. Hum Mov Sci. 2007;26:657–697. 10.1016/j.humov.2007.04.002 17604860

[23] Kelty-Stephen DG, Wallot S. Multifractality versus (mono-) fractality as evidence of nonlinear interactions across timescales: Disentangling the belief in nonlinearity from the diagnosis of nonlinearity in empirical data. Ecol Psychol. 2017;29:259–299.

[24] Booth CR, Brown HL, Eason EG, Wallot S, Kelty-Stephen DG. Expectations on hierarchical scales of discourse: Multifractality predicts both short- and long-range effects of violating gender expectations in text reading. Discourse Processes. 2018;55:12–30.

[25] Carver NS, Kelty-Stephen DG. Multifractality in individual honeybee behavior hints at colony-specific social cascades: Reanalysis of RFID data from five different colonies. Phys Rev E. 2017;95:022402 10.1103/PhysRevE.95.022402 28297945

[26] Carver NS, Bojovic D, Kelty-Stephen DG. Multifractal foundations of visually-guided aiming and adaptation to prismatic perturbation. Hum Mov Sci. 2017;55:61–72. 10.1016/j.humov.2017.07.005 28763703

[27] Hajnal A, Clark JD, Doyon JK, Kelty-Stephen DG. Fractality of body movements predicts perception of affordances: Evidence from stand-on-ability judgments about slopes. J Exp Psychol Hum Percept Perf. 2018;44:836–841. 10.1037/xhp0000510 29809050

[28] Kelty-Stephen DG, Dixon JA. Interwoven fluctuations in intermodal perception: Fractality in head-sway supports the use of visual feedback in haptic perceptual judgments by manual wielding. J Exp Psychol Hum Percept Perf. 2014;40:2289–2309. 10.1037/a0038159 25328996

[29] Kelty-Stephen DG, Stirling LA, Lipsitz LA. Multifractal temporal correlations in circle-tracing behaviors are associated with the executive function of rule-switching assessed by the Trail Making Test. Psychol Assess. 2016;28:171–176. 10.1037/pas0000177 26053002

[30] Nonaka T, Bril B. Fractal dynamics in dexterous tool use: The case of hammering behavior of bead craftsmen. J Exp Psychol Hum Percept Perf. 2014;40:218–231. 10.1037/a0033277 23875576

[31] O’Brien B, Wallot S. Silent reading fluency and comprehension in bilingual children. Front Psychol. 2016;7:1265 10.3389/fpsyg.2016.01265 27630590 PMC5005424

[32] Palatinus Z, Dixon JA, Kelty-Stephen DG. Fractal fluctuations in quiet standing predict the use of mechanical information for haptic perception. Ann Biomed Eng. 2013;41:1625–1634. 10.1007/s10439-012-0706-1 23188561

[33] Stephen DG, Arzamarski R, Michaels CF. The role of fractality in perceptual learning: Exploration in dynamic touch. J Exp Psychol Hum Percept Perf. 2010;36:1161–1173. 10.1037/a0019219 20718566

[34] Stephen DG, Hajnal A. Transfer of calibration between hand and foot: Functional equivalence and fractal fluctuation. Attent Percept Psychophys. 2011;73:1302–1328. 10.3758/s13414-011-0142-6 21598066

[35] Teng DW, Eddy CL, Kelty-Stephen DG. Non-visually-guided distance perception depends on matching torso fluctuations between training and test. Attent Percept Psychophys. 2016;78:2320–2328. 10.3758/s13414-016-1213-5 27739017

[36] Wallot S, O’Brien B, Coey CA, Kelty-Stephen DG. Power-law fluctuations in eye movements predict text comprehension during connected text reading. In: Noelle DC, Dale R, Warlaumont AS, Yoshimi J, Matlock T, Jennings CD, et al., editors. Proceedings of the 37th annual meeting of the Cognitive Science Society. Austin, TX: Cognitive Science Society;. 2015. pp. 2583–2588.

[37] Wallot S, O'Brien B, Haussmann A, Kloos H, Lyby MS. The role of reading time complexity and reading speed in text comprehension. J Exp Psychol Learning Mem Cognit. 2014;40:1745–1765. 10.1037/xlm0000030 24999710

[38] Ward RW, Kelty-Stephen DG. Bringing the nonlinearity of the movement system to gestural theories of language use: Multifractal structure of spoken English supports the compensation for coarticulation in human speech perception. Front Physiol. 2018;9:1152 10.3389/fphys.2018.01152 30233386 PMC6129613

[39] Bernstein NA. The coordination and regulation of movements Oxford: Pergamon; 1967 pp. 1–196.

[40] Latash ML. Synergy New York: Oxford University Press; 2008, pp. 1–432.

[41] Kelso JAS. Dynamic patterns: The self-organization of brain and behavior Cambridge, MA: MIT Press; 1995 pp. 1–334.

[42] Stephen DG, Dixon JA, Isenhower RW. Dynamics of representational change: Entropy, action, and cognition. J Exp Psychol Hum Percept Perf. 2009;35:1811–1832. 10.1037/a0014510 19968438

[43] Schvanefeldt RW. Finding meaning in psychology In: Healy AF, editor. Experimental cognitive psychology and its applications: Festschrift in honor of Lyle E. Bourne Jr., Walter Kintsch, and Thomas Landauer. Washington, DC: American Psychological Association; 2004 pp. 211–224.

[44] Fodor JA. The mind doesn't work that way: The scope and limits of computational psychology Cambridge, MA: MIT Press; 2000 pp. 1–144.

[45] Bußmann H. Lexikon der Sprachwissenschaft Abingdon: Taylor & Francis; 1996 pp. 1–530.

[46] Landauer TK, Dumais ST. A solution to Plato's problem: The latent semantic analysis theory of acquisition, induction, and representation of knowledge. Psychol Rev. 1997;104:211–240.

[47] Schertzer D, Lovejoy S. Generalised scale invariance in turbulent phenomena. J Physicochem Hydrodyn. 1985;6:623–635.

[48] Shlesinger MF, Zaslavsky GM, Klafter J. Strange kinetics. Nature. 1993;363:31–37.

[49] Turcotte DL, Malamud BD, Guzzetti F, Reichenbach P. Self-organization, the cascade model, and natural hazards. Proc Natl Acad Sci USA. 2002;99:2530–2537. 10.1073/pnas.012582199 11875206 PMC128572

[50] Turing AM. The chemical basis of morphogenesis. Phil Trans R Soc London B. 1952;237:37–72.

[51] Stephen DG, Boncoddo RA, Magnuson JS, Dixon JA. The dynamics of insight: Mathematical discovery as a phase transition. Mem Cognit. 2009;37:1132–1149. 10.3758/MC.37.8.1132 19933457

[52] Lorenz EN. Deterministic nonperiodic flow. J Atmospher Sci. 1963;20:130–141.

[53] Marwan N, Romano MC, Thiel M, Kurths J. Recurrence plots for the analysis of complex systems. Phys Rep. 2007;438:237–239.

[54] Wallot S, Leonardi G. Analyzing multivariate dynamics using Cross-Recurrence Quantification Analysis (CRQA), Diagonal-Cross-Recurrence Profiles (DCRP), and Multidimensional Recurrence Quantification Analysis (MdRQA)–a tutorial in R. 2018; Manuscript submitted for publication. 10.3389/fpsyg.2018.02232 PMC6288366 30564161

[55] Thomasson N, Hoeppner TJ, Webber CL Jr, Zbilut JP. Recurrence quantification in epileptic EEGs.Phys Lett A. 2001;279:94–101.

[56] Trulla LL, Giuliani A, Zbilut JP, Webber CL Jr. Recurrence quantification analysis of the logistic equation with transients. Phys Lett A. 1996;223:255–260.

[57] Johnston T. Alien and possum: Hanging out New York, NY: Aladdin; 2003 pp 1–48.

[58] O’Brien B, Wallot S, Haussmann A, Kloos H. Using complexity metrics to assess silent reading fluency: A cross-sectional study comparing oral and silent reading. Sci Studies Reading. 2014;18:235–254.

[59] Brainard DH. The Psychophysics Toolbox. Spatial Vis. 1997;10:433–436. 9176952

[60] Wallot S. Recurrence quantification analysis of processes and products of discourse: A tutorial in R. Discourse Processes. 2017;54:382–405.

[61] Fraser AM, Swinney HL. Independent coordinates for strange attractors from mutual information. Phys Rev A. 1986;33:1134 10.1103/PhysRevA.33.1134 9896728

[62] Kennel MB, Brown R, Abarbanel HD. Determining embedding dimension for phase-space reconstruction using a geometrical construction. Phys Rev A. 1992;45:3403 10.1103/PhysRevA.45.3403 9907388

[63] Marwan N. CROSS RECURRENCE PLOT TOOLBOX 5.17 (R29.1). Retrieved September 19, 2018, from http://tocsy.pik-potsdam.de/CRPtoolbox/

[64] Singer JD, Willett JB, Applied longitudinal data analysis New York: Oxford University Press; 2003 Pp. 1–644.

[65] Kormos J, Babuder MK, Pizorn K. The role of low-level first language skills in second language reading, reading-while-listening, and listening performance: A study of young dyslexic and non-dyslexic language learners. Appl Linguistics. 2018; amy028 10.1093/applin/amy028

[66] Kim M, Crossley SA, Skalicky S. Effects of lexical features, textual properties, and individual differences on word processing times during second language reading comprehension. Read Writ. 2018;31:1155–1180.

[67] Elgort I, Brysbaert M, Stevens M, Van Assche E. Contextual word learning during reading in a second language. Studies Second Lang Acquisition. 2018;40:341–366.

[68] Cox TL, Bown J, Bell TR. In advanced L2 reading proficiency assessments, should the question language be in the L1 or the L2?: Does it make a difference? In: Winke P, Gass S, editors. Foreign language proficiency in higher education: Educational linguistics, vol. 37 Berlin: Springer; 2018 pp. 117–136.

[69] Verhoeven L, Voeten M, Vermeer A. Beyond the simple view of early first and second language reading: The impact of lexical quality. J Neurolinguistics. 10.1016/j.jneuroling.2018.03.002

[70] Hock HS, Kelso JAS, Schöner G. Bistability and hysteresis in the organization of apparent motion patterns. J Exp Psychol Hum Percept Perf. 1993:19:63–80. 10.1037//0096-1523.19.1.63 8440989

[71] Kelso JAS, Buchanan JJ, Murata T. Multifunctionality and switching in the coordination dynamics of reaching and grasping. Hum Mov Sci. 1994;13:63–94.

[72] Tuller B, Nguyen N, Lancia L, Vallabha GK. Nonlinear dynamics in speech perception In: Huys R, Jirsa VK, editors. Nonlinear dynamics in humana behavior: Studies in computational intelligence. Berlin: Springer; 2010 pp. 135–150.

[73] Prior A, MacWhinney B. A bilingual advantage in task switching. Bilingualism Lang Cognit. 2010;13:253–262. 10.1017/S1366728909990526 PMC9724810 36479004

[74] Declerck M, Grainger J, Koch I, Philipp AM. Is language control just a form of a executive control? Evidence for overlapping processes in language switching and task switching. J Mem Lang. 2017;95:138–145.

[75] Calabria M, Hernandez M, Branzi FM, Costa A. Qualitative differences between bilingual language control and executive control: Evidence from task-switching. Front Psychol. 2012;2:399 10.3389/fpsyg.2011.00399 22275905 PMC3257869

[76] Kiesel A, Steinhauser M, Wendt M, Falkenstein M, Jost K, Philipp AM, Koch I. Control and interference in task switching—A review. Psychol Bull. 2010;136:849–874. 10.1037/a0019842 20804238

[77] Waszak F, Hommel B, Allport A. Task-switching and long-term priming: Role of episodic stimulus-task bindings in task-shift costs. Cognit Psychol. 2003;46:361–413. 12809680 10.1016/s0010-0285(02)00520-0

[78] Koch I, Gade M, Schuch S, Philipp AM. The role of inhibition in task switching: A review. Psychonomic Bull Rev. 2010;17:1–14. 10.3758/PBR.17.1.1 20081154

[79] Vandierendonck A, Liefooghe B, Verbruggen F. Task switching: Interplay of reconfiguration and interference control. Psychol Bull. 2010;136:601–626. 10.1037/a0019791 20565170

[80] Koch I, Poljac E, Mueller H, Kiesel A. Cognitive structure, flexibility, and plasticity in human multitasking—An integrative review of dual-task and task-switching research. Psychol Bull. 2018;144:557–583. 10.1037/bul0000144 29517261

[81] Liefooghe B. The contribution of task-choice response selection to the switch cost in voluntary task switching. Acta Psychologica. 2017;178:32–40. 10.1016/j.actpsy.2017.05.006 28570859

[82] Huff MJ, Balota DA, Minear M, Aschenbrenner AJ, Duchek JM. Dissociative global and local task-switching costs across younger adults, middle-aged adults, older adults, and very mild Alzheimer’s disease individuals. Psychol Aging. 2015;30:727–739. 10.1037/pag0000057 26652720 PMC4681312

[83] Stasenko A, Matt GE, Gollan TH. A relative bilingual advantage in switching with preparation: Nuanced explorations of the proposed association between bilingualism and task switching. J Exp Psychol Gen. 2017;146:1527–1550. 10.1037/xge0000340 28714710 PMC5668152

[84] Kleinman D, Gollan TH. Inhibition accumulates over time at multiple processing levels in bilingual language control. Cognit. 2018;173:115–132. 10.1016/j.cognition.2018.01.009 PMC5812452 29405945

[85] Meuter RFI, Allport A. Bilingual language switching in naming: Asymmetrical costs of language selection. J Mem Lang. 1999;40:25–40.

[86] Rayner K, Pollatsek A. The psychology of reading New York: Routledge; 1989 pp. 1–544.

