# Supplementary material for: Switching between reading tasks leads to phase-transitions in reading times in L1 and L2 readers
Source: PLoS One. 2019 Feb 5;14(2):e0211502. doi: 10.1371/journal.pone.0211502 (PMC6363172; doi:10.1371/journal.pone.0211502)
Supplement: S1 File — (DOCX) [file pone.0211502.s001.docx]

**Appendix A**

Overview over the AIC-plots for different sizes of the phase-transition parameters in number of words for the R🡪O (Figure 7) and the O🡪R (Figure 8) condition for study 1. We selected the model for lowest AIC, spaning 190 words in the R🡪O condition and 160 words in the O🡪R condition.

**Fig 1. AIC-plot for the H3-models of the O🡪R condition in study 2 as a function of varying the length of the predictor for the phase-transition period.** As can be seen, three variables (*%LAM*, *TT*, and *maxV*) show a minimum at length of 190 for the phase-transition between the connected text reading task and the random word reading task, while recurrence rate (*%REC*) shows a somewhat higher value, namely 240. Because *%LAM*, *TT*, and *maxV* are in agreement with each other, we chose 190 words as the length for the transition period.

**Fig 2. AIC-plot for the H3-models of the R🡪O condition in study 1 as a function of varying the length of the predictor for the phase-transition period.** As can be seen, three variables (%REC, *%LAM*, and *TT*) show a minimum at length of 160 for the phase-transition between the random word reading task and the connected text reading task, while the maximum vertical line length (*TT*) shows a somewhat higher value, namely 200. Because *%REC*, *%LAM*, and *TT* are in agreement with each other, we chose 160 words as the length for the transition period.
