# Supplementary material for: Switching between reading tasks leads to phase-transitions in reading times in L1 and L2 readers
Source: PLoS One. 2019 Feb 5;14(2):e0211502. doi: 10.1371/journal.pone.0211502 (PMC6363172; doi:10.1371/journal.pone.0211502)
Supplement: S2 File — (DOCX) [file pone.0211502.s002.docx]

**Appendix B**

Overview over the AIC-plots for different sizes of the phase-transition parameters in number of words for the R🡪O (Figure 9) and the O🡪R (Figure 10) condition for study 2. We selected the model for lowest AIC, spanning 120 words in the R🡪O condition and 50 words in the O🡪R condition.

**Fig 1. AIC-plot for the H3-models of the O🡪R condition in study 2 as a function of varying the length of the predictor for the phase-transition period.** As can be seen, three variables (*%LAM*, *TT*, and *maxV*) show a minimum at length of 50 for the phase-transition between the connected text reading task and the random word reading task, while recurrence rate (*%REC*) shows a somewhat higher value, namely 90. Because *%LAM*, *TT*, and *maxV* are in agreement with each other, we chose 50 words as the length for the transition period.

**Fig 2. AIC-plot for the H3-models of the R🡪O condition in study 1 as a function of varying the length of the predictor for the phase-transition period.** As can be seen, all four variables show a minimum at lengths 110 or 120 for the phase-transition between the random word reading task and the connected text reading task. Hence, we chose 120 words as the length for the transition period.
