# Supplementary material for: Switching between reading tasks leads to phase-transitions in reading times in L1 and L2 readers
Source: PLoS One. 2019 Feb 5;14(2):e0211502. doi: 10.1371/journal.pone.0211502 (PMC6363172; doi:10.1371/journal.pone.0211502)
Supplement: S3 File — (DOCX) [file pone.0211502.s003.docx]

**Description of the data set**

The data set accompanying the article “Switching between reading tasks leads to phase-transitions in reading times in L1 and L2 readers” is provided as a CSV-file in person-period format and contains the following columns:

study: coding for the two studies in this data set (“1” = study 1; “2” = study 2)

condition: coding for the two conditions (orders in which stimuli were presented) in each of the two studies (“1” = O🡪R; “2” = R🡪O)

participant: coding for the participant number as a running count across studies (ranging from “1” to “60”)

time: coding for the order in which reading times were collected within each participant (ranging from “1” to “1082”)

data: variable containing the reading times for each word (in seconds)

ratingO: disaggregated meaingfulness ratings for the ordered text in study 2 (ranging from “1”, meaingless, to “7”, very meaningful; the value of “-1” indicates that no such ratings were collected for participants in study 1)

ratingR: disaggregated meaingfulness ratings for the random word list in study 2 (ranging from “1”, meaingless, to “7”, very meaningful; the value of “-1” indicates that no such ratings were collected for participants in study 1)
